# Insights into genetic diversity and phenotypic variations in domestic geese through comprehensive population and pan-genome analysis

**DOI:** 10.1186/s40104-023-00944-y

**Published:** 2023-11-24

**Authors:** Guangliang Gao, Hongmei Zhang, Jiangping Ni, Xianzhi Zhao, Keshan Zhang, Jian Wang, Xiangdong Kong, Qigui Wang

**Affiliations:** 1 Chongqing Academy of Animal Science, Rongchang District, Chongqing, 402460 China; 2 Livestock and Poultry Multi-Omics Key Laboratory of Ministry of Agriculture and Rural Affairs, College of Animal Science and Technology, Sichuan Agricultural University, Chengdu, 611130 China; 3 Chongqing Engineering Research Center of Goose Genetic Improvement, Rongchang District, Chongqing, 402460 China; 4 Department of Cardiovascular Ultrasound and Non-Invasive Cardiology, Sichuan Academy of Medical Sciences and Sichuan Provincial People’s Hospital，University of Electronic Science and Technology of China, Chengdu, 611731, China; 5 Ultrasound in Cardiac Electrophysiology and Biomechanics Key Laboratory of Sichuan Province, University of Electronic Science and Technology of China, Chengdu, 611731, China; 6 JiguangGene Biotechnology Co., Ltd., Nanjing, 210032, China; 7 Jiangsu Agri-Animal Vocational College, Taizhou, 225300, China; 8 Present Address: Poultry Science Institute, Chongqing Academy of Animal Science, No. 51 Changzhou Avenue, Rongchang District, Chongqing, 402460 P. R. China

**Keywords:** Gene-CDS haplotype, Goose, GWAS, Pan-genome, Presence-absence variation, Selection signal

## Abstract

**Background:**

Domestic goose breeds are descended from either the Swan goose (*Anser cygnoides*) or the Greylag goose (*Anser anser*), exhibiting variations in body size, reproductive performance, egg production, feather color, and other phenotypic traits. Constructing a pan-genome facilitates a thorough identification of genetic variations, thereby deepening our comprehension of the molecular mechanisms underlying genetic diversity and phenotypic variability.

**Results:**

To comprehensively facilitate population genomic and pan-genomic analyses in geese, we embarked on the task of 659 geese whole genome resequencing data and compiling a database of 155 RNA-seq samples. By constructing the pan-genome for geese, we generated non-reference contigs totaling 612 Mb, unveiling a collection of 2,813 novel genes and pinpointing 15,567 core genes, 1,324 softcore genes, 2,734 shell genes, and 878 cloud genes in goose genomes. Furthermore, we detected an 81.97 Mb genomic region showing signs of genome selection, encompassing the *TGFBR2* gene correlated with variations in body weight among geese. Genome-wide association studies utilizing single nucleotide polymorphisms (SNPs) and presence-absence variation revealed significant genomic associations with various goose meat quality, reproductive, and body composition traits. For instance, a gene encoding the SVEP1 protein was linked to carcass oblique length, and a distinct gene-CDS haplotype of the *SVEP1* gene exhibited an association with carcass oblique length. Notably, the pan-genome analysis revealed enrichment of variable genes in the “hair follicle maturation” Gene Ontology term, potentially linked to the selection of feather-related traits in geese. A gene presence-absence variation analysis suggested a reduced frequency of genes associated with “regulation of heart contraction” in domesticated geese compared to their wild counterparts. Our study provided novel insights into gene expression features and functions by integrating gene expression patterns across multiple organs and tissues in geese and analyzing population variation.

**Conclusion:**

This accomplishment originates from the discernment of a multitude of selection signals and candidate genes associated with a wide array of traits, thereby markedly enhancing our understanding of the processes underlying domestication and breeding in geese. Moreover, assembling the pan-genome for geese has yielded a comprehensive apprehension of the goose genome, establishing it as an indispensable asset poised to offer innovative viewpoints and make substantial contributions to future geese breeding initiatives.

**Supplementary Information:**

The online version contains supplementary material available at 10.1186/s40104-023-00944-y.

## Background

Domestic goose is a highly valued waterfowl widely raised because of its economic benefits. Previous studies on fossils and genomic sequences have indicated that Chinese indigenous geese, except the Yili goose, were primarily domesticated from Swan geese (*Anser cygnoides*) [1]. In contrast, European geese were predominantly domesticated from Greylag geese (*Anser anser*). The domestication process of geese can be traced back to over 7,000 years ago [2–4]. Goose eggs, liver, and meat are nutritious and favored by humans. Goose feathers are valuable raw materials for industrial production [5]. Due to these features, humans have domesticated geese for various purposes, which has led to the development of many indigenous goose breeds. During domestication, the migratory Swan and Greylag goose were developed into domestic geese with accelerated growth rates, extended laying periods, and increased reproductive capacity. For instance, Swan geese typically lay 5 to 8 eggs annually, weigh between 2.8 to 3.5 kg, and possess white feathers [6], while Chinese goose breeds domesticated from Swan geese display significant variations in egg production, body weight, and feather coloration, with noteworthy examples being the Zi goose breed, which boasts an average annual egg yield of 93 eggs per individual, and the Lion head goose, with an average body weight of 13.55 kg, along with pure white-feathered breeds like the Sichuan white goose [6, 7].

The advances in sequencing technology and bioinformatics have facilitated the genetic study of various traits in geese. Over the years, reference genomes for numerous goose breeds have been assembled and published. Following the successful assembly of the Zhedong white goose genome [8], genomes of several other goose breeds, including the Sichuan white goose, Tianfu goose, wild Swan goose, Xingguo gray goose, and Lion head goose, have also been published [7–14]. The availability of these genome sequences allows the analysis of the genetic basis of differences in traits (such as the variation in reproductive capacity and body weight). For example, the genomic analysis of Xingguo gray geese and Gang geese revealed a natural genetic mutation: a 14-bp insertion in the endothelin receptor B subtype 2 (*EDNRB2*) gene responsible for white feathers in Chinese indigenous goose [11, 15]. Previous studies have identified SNPs near or within genes associated with geese reproductive ability [16, 17].

Because of the availability of high-quality reference genomes, large-scale whole-genome sequencing (WGS) and transcriptome sequencing have become essential approaches to studying the evolution and domestication of geese. The WGS data from wild and domestic geese revealed that many modern European goose breeds share a significant (> 10%) ancestry with Chinese indigenous goose, and the frequent gene exchange has been confirmed since domestic and wild geese divergence 5,300 generations ago [18]. In a previous study, the WGS data from 990 geese were analyzed, which revealed that the genetic diversity of Chinese geese was higher than that of European geese, and a specific haplotype cluster was identified that distinguished white and grey geese [11]. A combination of morphological, transcriptomic, and genomic resequencing studies on Chinese indigenous goose identified 17 and 21 candidate genes associated with knob formation in the skin and bones, respectively, including iodothyronine deiodinase 2 (*DIO2*), that play a crucial role in determining goose knob phenotype [19]. Chinese indigenous goose breeds from different regions exhibit distinct traits in body weight, reproduction ability and feather color. For instance, Wen et al. [20] reported that an 18-bp deletion in Receptor tyrosine kinase (*KIT*) is strongly associated with white feathers in 18 Chinese indigenous white and grey geese. Moreover, the WGS data from diverse populations exhibiting variations in meat quality, growth traits, and reproduction traits have revealed a set of candidate genes associated with a wide range of characteristics, including the gene DEAH-box polypeptide 15 (*DHX15*) related to meat quality, LIM domain binding 2 (*LDB2*), Slit guidance ligand 2 (*SLIT2*), and recombination signal binding protein for immunoglobulin kappa J region (*RBPJ*) are associated with growth traits, and the gene potassium voltage-gated channel interacting protein 4 (*KCNIP4*) is implicated in reproduction traits [21]. These results are of great significance for understanding the domestication process of geese and providing guidance for breeding.

With appropriate experimental designs, RNA-seq of various organs or growth stages of various goose breeds can further reveal the occurrence and regulation of various goose traits. The Lion head goose is a large-bodied goose in China. Transcriptome analysis of myoblast proliferation and differentiation in Lion head geese revealed that differentially expressed genes at various developmental stages were mainly involved in the Wnt signaling pathway, revealing the potential regulatory role of this pathway in muscle growth in Lion head geese [22]. Light significantly influences both the growth rate and quality of poultry; an RNA-seq analysis of Zhedong white geese’s leg muscle subjected to extended and abbreviated light cycles over 60 d unveiled the prominent impact of light on the PI3K-Akt signaling pathway [23]. Reproductive ability is one of the crucial traits of geese. Transcriptome analysis comparing geese with high and low reproductive ability revealed that members of the 5-hydroxytryptamine gene family regulate ovarian metabolic function to affect reproductive ability [24]. These extensive genomic and transcriptomic studies provided a vital reference for a deeper understanding of various molecular mechanisms of geese.

Relying solely on a single reference genome is inadequate for fully representing the diverse range of genetic sequences within a species; however, constructing a pan-genome consisting of multiple individuals from the same species has proven highly effective in addressing this limitation [25]. Recent pan-genomic studies have revealed the substantial impact of genome-wide structural variations (SV) or presence-absence variation (PAV) on animal traits [25–28]. In chicken, the pan-genomic study identified 66.5 Mb of novel sequences not present in the reference genome and predicted 4,063 highly credible, new protein-coding genes; more importantly, through population-level PAV analysis, this study identified a deletion variant in the insulin-like growth factor 2 mRNA-binding protein 1 (*IGF2BP1*) gene promoter region that affects chicken body size [29]. This highlighted the critical role of pan-genomic analysis in genomic research and breeding of domestic poultry. Apart from poultry, pan-genomic analysis of other domestic animals has provided important information on breeding. In a study involving 12 pig genomes, researchers identified 72.5 Mb of novel sequences absent from the reference genome and discovered a Chinese pig-specific gene known as tazarotene-induced gene 3 (*TIG3*), which plays a crucial role in regulating fatty acid metabolism [30]. Cattle are an important livestock worldwide. After assembling and aligning the resequencing sequences of 898 cattle from 57 accessions, 83 Mb of the sequence was missing from the reference genome, and in some of the variants with nucleotide insertion, essential functional genes were affected [31].

To comprehensively understand the genetic diversity and molecular mechanisms underlying phenotypic variations in geese, this study conducted comprehensive pan-genome using multiple reference goose genomes (Tianfu goose, Sichuan white goose, and Zhedong white goose) on 659 WGS data from 647 individual geese and 155 RNA-seq datasets. Based on the Tianfu goose genome sequence, we performed a comparative genomic analysis between Chinese and European geese to reveal genomic distinctions and identify genes exhibiting significant frequency differences related to economic traits and feather color. To identify molecular markers associated with economic traits (meat quality, body size, reproduction, and egg quality traits), we conducted genome-wide association studies based on SNPs and gene PAV data from Sichuan white goose population (a Chinese indigenous goose renowned for its dual-purpose role in both meat and egg production). This work will provide insight into the molecular mechanisms underlying genetic diversity and phenotypic variability in geese during domestication and breeding, offering valuable resources to facilitate genetic research and breeding in geese.

## Methods

### Experimental animals

In this study, a total of 659 WGS datasets were utilized to construct the goose pan-genome. Within this dataset, 378 WGS datasets were generated as part of our study, including 9 goose breeds (Table S1 for the list of included breeds: 25 Huoyan geese, 20 Lion head geese, 24 Magang geese, 215 Sichuan white geese, 20 Taihu geese, 15 Xupu geese, 24 Zi geese, 20 Landes geese and 15 White Roman geese). Additionally, we downloaded an additional 281 WGS datasets from a publicly accessible database (Table S2, which includes data on the 209 Sichuan white goose and 72 individuals from wild goose breeds) [16, 32–34]. In conclusion, there are 7 Chinese indigenous goose breeds (*Anser cygnoides domestica*, including Huoyan geese, Lion head geese, Magang geese, Sichuan white geese, Taihu geese, Xupu geese, and Zi geese), two European domestic goose breeds (*Anser anser domestica*, including Landes geese and White Roman geese) and wild goose species.

We collected 378 blood samples from the medial pterygoid vein of the geese using vacuum tubes containing EDTA to prevent coagulation. These samples were sourced from two primary locations: the AnFu Waterfowl Breeding Base in Chongqing City and the National Waterfowl Germplasm Resource Gene Pool in Taizhou, China. These collected samples were then preserved at −20 °C in a freezer. Genomic DNA extraction was performed by isolating it from the whole blood using a DNA extraction kit (DP332; Tiangen Biotech, Beijing, China). Subsequently, the concentration and quality of the extracted DNA were assessed using a Nano Vue spectrophotometer (Cytiva Life Sciences, Marlborough, MA, USA) and agarose gel electrophoresis. The WGS libraries for these 378 individuals were prepared in accordance with the standard Illumina library preparation kit protocol (Illumina, San Diego, CA, USA). After library preparation, we conducted WGS on the Illumina HiSeq X Ten platform at Novogene Biotechnology Corporation in Beijing, China.

The classification of the 7 Chinese indigenous goose breeds was determined based on factors including body weight, reproductive capacity, and feather color. This classification was derived from data provided by the China Goose Genetic Resources Database, accessible at http://www.yzcom.com/webdemo/goose. According to the previous study [35], the Chinese indigenous geese were categorized according to their reproductive ability, classify into high (Huoyan and Zi goose), middle (Sichuan white and Taihu goose), and low (Lion head, Magang, and Xupu goose) groups. Additionally, the geese were further categorized based on their body weight: heavy (Lion head goose), middle (Sichuan white and Xupu goose), and low (Huoyan, Taihu, and Zi goose) groups. Furthermore, the feather color of the geese was classified as either white (Sichuan white, Huoyan, Xupu, Zi, and Taihu goose) or gray (Magang and Lion head goose) groups.

### Phenotypic traits in Sichuan white geese (*Anser cygnoides domestica*)

In this study, we obtained body size and meat quality traits from 70-day-old male Sichuan white geese population (215 individuals), while reproductive and egg quality phenotype data from 1-year-old female geese population (209 individuals), which were provided by the AnFu Waterfowl Breeding Base in Chongqing City. All of the collected phenotypic data were subsequently utilized in SNP-GWAS and PAV-GWAS analyses. For body size and meat quality traits, 215 70-day-old male Sichuan white geese were randomly selected from a shared incubation batch and reared under controlled conditions for the study. After blood sampling, we conducted assessments encompassing a range of morphometric measurements, including body length, carcass femur length, carcass keel bone length, chest depth, chest width, feet weight, keel bone length, keel length, leg circumference, neck length, and tibia length. Subsequently, carcass characteristics, such as the weight of semi-eviscerated carcass, eviscerated carcass, subcutaneous fat, as well as meat quality of thigh muscle and breast muscle, were evaluated. Additionally, the weight of visceral organs, including heart, liver, kidney, lung, spleen, pancreas, gizzard, and proventriculus was quantified. After slaughter, breast muscle samples from the right side were collected precisely 2 h later. Furthermore, a portable pH meter (pHCore-kit, Sartorius Lab Instruments GmbH, Goettingen, Germany) was employed with its glass electrode inserted directly into the muscle following calibration using buffers (pH 4.01), and the pH values were calculated from the average of three measurement points. The study involved the analysis of various meat quality parameters, including lightness (L*), redness (a*), yellowness (b*) of meat color, pH value, shear force, cooking loss rate, and crude fat content.

For egg-laying and reproduction traits, our previous study involved the individual rearing of each female Sichuan white goose within the population, starting from birth and continuing until the non-laying period, which extended for 66 weeks [16]. These geese were housed in separate cages (600 mm × 800 mm × 900 mm) throughout their egg-laying period, spanning weeks 28 to 66. We diligently collected and marked eggs from each goose daily. Detailed records were kept for various parameters of each individual, including birth body weight, 70-day body weight, body weight at first egg laying, egg number at 48 weeks [16]. Additionally, during weeks 35 to 40, we collected three consecutive eggs from each goose and determined egg weight, egg yolk color, egg relative density, egg shell strength, egg shell thickness, egg shell weight, egg yolk weight, egg index traits. Moreover, we introduced healthy male geese from a separate colony at a 1:4 male-to-female ratio for mating with the female geese. Finally, daily candling was performed on all eggs to determine the fertility, qualified egg rate, plasma concentrations of progesterone (P), follicle-stimulating hormone (FSH), prolactin (PRL) and oestrogen (E2) [36].

### SNP calling and population genomic analysis

For all of the 659 WGS data, regardless of whether it was generated in our laboratory or obtained from public databases, we conducted quality control, trimming, and filtering of raw sequencing data using the methodologies detailed in a previous study [16]. Subsequently, the filtered WGS data from all geese were meticulously aligned to the goose reference genome (version ASM1303099v1) using the Burrows-Wheeler Alignment (BWA) software [37]. Potential PCR duplicates were identified and marked using the “MarkDuplicates” tool in GATK software (version 4.2.6.1). SNP calling was conducted on the GVCF file using HaplotypeCaller in GATK. The called SNPs then underwent quality filtering using VariantFiltration, applying the following parameters: –filter-expression “QD < 2.0 || FS > 60.0 || MQ < 40.0 || SOR > 3.0 || MQRankSum <  − 12.5 || ReadPosRankSum <  − 8.0” –filter-name “snp_filter” –genotype-filter-expression “DP < 2 || DP > 50” –genotype-filter-name “dp_fail”. Finally, VCFtools [38] was used to remove sites with missing rate > 90% and allele frequency < 5%. SNP annotation was performed using variant SnpEff [39]. The fixation index (*F*st) between different populations was calculated through VCFtools. An IQ-TREE analysis (default parameters) was employed to construct the phylogenetic tree of geese based on SNP data [40]. ADMIXTURE (default parameters) was utilized to analyze the population structure of geese using SNP data [41]. Investigating gene flow among distinct populations was achieved using Treemix (default parameters) [42]. Nucleotide diversity (Pi) for different populations was computed using VCFtools (default parameters) [43].

### Selective signal analysis between geese groups

We identified selective sweep regions on chromosomes using the Tianfu goose genome as a reference. An XP-CLR test (updated Python version released on https://github.com/hardingnj/xpclr) was performed to detect selective signals between various goose populations [44]. Each chromosome was independently analyzed and divided into nonoverlapping windows of 10 kb. The average XP-CLR likelihood scores of each window were calculated. Regions with XP-CLR, a likelihood score average in the top 5% of the entire genome, were defined as having strong selective signals. For the regions with XP-CLR likelihood scores in the top 20%, adjacent regions or regions separated by one window were merged into a new window. The maximum average XP-CLR likelihood score among these regions was taken as the XP-CLR likelihood score for the new window, and the maximum average XP-CLR likelihood score was used as the XP-CLR likelihood score of this new window. In the regions of candidate genes and their 5 kb upstream and downstream, the *F*st values between various goose populations were calculated.

### Pan-genome construction

Based on the previous step, unmapped reads or low-quality mapping sequences were extracted using SAMtools v1.9 (SAMtools fastq -f 12, SAMtools fastq -f 68 -F 8, and SAMtools fastq -f 132 -F 8) [45]. The unmapped reads of each individual were assembled using MaSuRCA [46], and contigs smaller than 500 bp were removed. However, poorly mapped reads might have been still present in the unmapped reads extracted using SAMtools, and contigs identical to the reference genome might still have been present in the assembled contigs. Therefore, the remaining contigs were aligned to the goose reference genome and mitochondrial genome using nucmer [47] from the Mummer software package. If a contig had a region larger than 300 bp that could be aligned to the reference genome with over 90% similarity, this region was considered a reliable alignment region. Contigs that did not have such reliable alignment regions were defined as unaligned contigs or fully unaligned contigs. In the sequences that could be aligned to the reference genome, if there were regions larger than 500 bp with less than 90% similarity to the reference genome, these regions were extracted as partially unaligned contigs.

The sequences of fully unaligned contigs and partially unaligned contigs were merged and subjected to redundancy removal using CD-HIT-EST software [48]. To further eliminate redundancy, the resulting nonredundant sequences were subjected to all-vs-all comparison using blastn and nucmer, and the comparison results were processed using an in-house Perl script, with a threshold of 90% similarity over 90% of the region for further redundancy removal. The final nonredundant sequences were compared with the NT database using blastn, and sequences belonging to archaea, viruses, bacteria, fungi, and Viridiplantae were removed based on the species information in the comparison results. In addition, Kraken2 was used to annotate the new sequences against the Kraken2-microbial database, and sequences annotated as microorganisms were removed [49]. Finally, the sequences obtained in the previous step were compared to the reference genome using blastn to ensure that these new sequences were not present in the reference genome. Nucmer was used to compare the reference genome and filter the sequences using the same criteria as above, resulting in nonredundant, uncontaminated, and non-reference sequences.

Considering the differences between the reference genome of the Tianfu goose used in this study and other versions of reference genomes, Zhedong white goose [8] and Sichuan white goose [9] were downloaded and compared with the reference genome. ppsPCP [50] was used to extract PAV sequences in the genomes of the Zhedong white goose and Sichuan white goose but not in the Tianfu genome. PAV genes were defined as those with more than 80% overlap with the corresponding genomic regions in the two genomes, as per the standard set by a pan-genome study on *Brassica napus* [51]. Further, the non-reference sequences constructed based on the second-generation resequencing data were compared with the PAV sequences obtained from inter-genome comparisons using blastn. The get_coverage_filter.pl script in ppsPCP was used to remove non-reference sequences highly similar to the identified PAV sequences. Finally, the PAV sequences obtained from multi-genome comparisons (Tianfu goose, Sichuan white goose, and Zhedong white goose), and non-reference sequences assembled from WGS data were merged to form the pan-genome of geese.

### Pan-genome annotation

For pan-genome annotation, we downloaded 155 RNA-seq datasets from the GEO or SRA database spanning ten distinct tissue types [52, 53]. Based on the RepBase (v17.01, http://www.girinst.org/repbase) transposon repeat sequence library, RepeatMasker was used to annotate repetitive sequences in the novel contigs of geese [54]. Additionally, RepeatModeler was used to construct a de novo repeat sequence library for the new goose sequences, which was further used by RepeatMasker for further annotation [55]. Tandem Repeats Finder was used to annotate tandem repeat sequences in the novel contigs of geese [56]. Hisat2 [57] was used to map RNA-seq data to the novel contigs, and unmapped reads were extracted using SAMtools. Trinity [58] was used for the de novo assembly of these unmapped reads, followed by redundancy reduction using cd-hit-est. Finally, maker2 [59] was used to integrate gene structure predictions from Augustus [60] (trained on the reference genome), transcripts assembled from RNA-seq, and protein sequences from the reference genome to predict gene structures in the novel contigs. Gene annotations were compared with repeat sequence annotations, and genes with overlap > 50% with repeat regions were removed.

### PAV selection analysis

The resequencing data of geese were mapped to the pan-genome sequence using bowtie2 [61]. The presence and absence of genes in the pan-genome were identified using SGSGeneLossv0.1 software [62], with the parameters minCov = 2 and lostCutoff = 0.2 (that is, a gene was considered present if at least 2 reads covered at least 20% of the region covered by its exons). Based on the binary gene PAV data, a maximum-likelihood phylogenetic tree (1,000 bootstraps) was constructed using iqtree [63]. Moreover, the population structure of geese was analyzed using STRUCTURE [64] based on the PAV data. In addition, 1, 1–2, and 2–3 kb upstream regions were used as the gene promoter regions in geese. The PAV in the promoter regions was detected using the same criteria for identifying PAV in the genes.

This study categorized the geese based on distinct ancestors (Swan and Greylag goose) and various phenotypic traits, including body weight, egg production, and feather color characteristics. We employed Fisher’s exact test to identify differences in gene frequencies among the different goose populations. The resulting *P*-values were then adjusted using the Benjamini–Hochberg method, with the threshold set at FDR < 0.001 and a frequency difference > 2. We conducted hypergeometric tests to analyze the genes with different frequencies for enrichment in both GO and KEGG categories, utilizing an adjusted *P*-value threshold of 0.05 for significance.

### SNP-GWAS and PAV-GWAS in Sichuan white geese (*Anser cygnoides domestica*)

We used binary PAV data and SNP to analyze GWAS using FarmCPU (default parameters) [65]. Since the number of PAVs is smaller than that of SNPs, we performed principal component analysis (PCA) analysis based on SNP information using GCTA [66]. The PCA results were used as covariates for SNP and gene PAV-GWAS analysis. The significance threshold was set at 0.05/SNP and 0.05/PAV numbers. Using the CandiHap software package [67] with default settings, haplotypes were derived from SNPs within each candidate gene’s 1-kb upstream to 500-bp downstream regions. Subsequently, the associations between these haplotypes and phenotypes were analyzed.

### Retrieval of RNA-seq data and expression analysis

RNA-seq data was downloaded from several organs or tissues of geese, including abdominal adipose tissue, granulosa cells, hypothalamus, liver, ovarian stroma, ovary, pituitary, skin, and subcutaneous adipose tissue (NCBI bioproject numbers PRJNA489234, PRJNA549469, PRJNA552525, PRJNA598883, PRJNA615385, PRJNA674406, PRJNA699919, PRJNA705645, and PRJNA825140, respectively). Clean data were mapped to the genome using Hisat2, and the reads of each gene were counted using featureCounts [68]. FPKM (Fragments per kilobase of transcript per million mapped reads) values and CPM (counts per million) values were calculated to assess the gene expression. We performed PCA analysis based on the expression values. We employed DESeq2 software to identify the differential expression analysis of genes [69, 70]; *Q*-values < 0.05 were considered significantly differentially expressed genes. For the RNA-seq data, which were sampled and sequenced at multiple time points, differential expression analysis was performed using maSigPro [69], a software designed for time series data. The threshold for differential expression was set at FDR < 0.05. The variation types of the differentially expressed genes identified were analyzed using vep [71]. The tissue specificity index (TAU) was calculated using the following formula.

$$tau=\frac{n}{n-1}-\frac{{\sum }_{i=1}^{n}{x}_{i}}{\left(n-1\right)\times \underset{1\le i\le n}{\mathrm{max}}\left({x}_{i}\right)}$$

where *n* represents the number of groups, *x* represents the mean expression value of genes in various groups, and *i* represents one of the groups. The TAU ranges from 0 (broad expression) to 1 (specific expression). A hypergeometric distribution test was used for genes with specific expressions to perform enrichment analysis of GO terms.

## Results

### Population structure analysis across nine goose populations

**Fig. 1:**
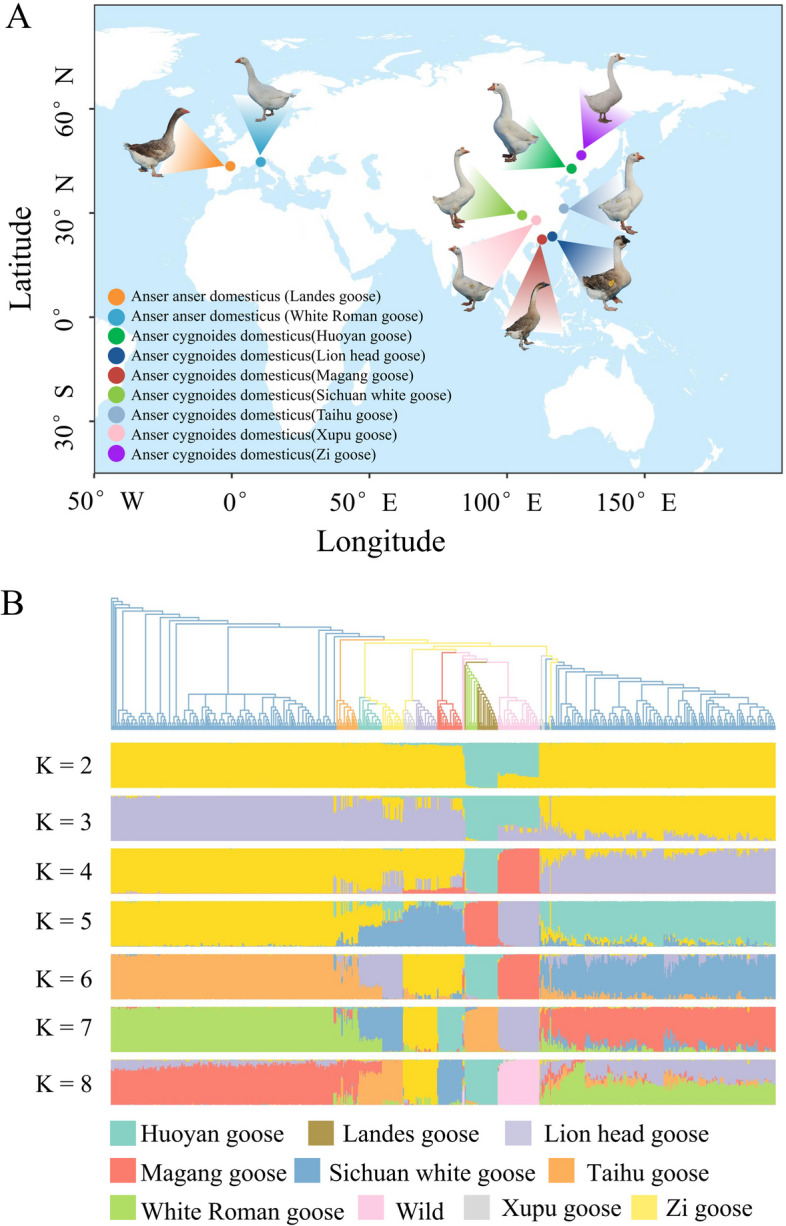
Sampling and population analysis of geese using whole-genome sequencing (WGS) data. **A** Distribution of the goose breeds used in this study (It is important to emphasize that all goose samples, including those of the Landes goose and White Roman goose, were exclusively collected in China). The color of the dots indicates the name of the goose breed. **B** The phylogenetic tree and population structure analysis of geese based on the whole genome SNP sites. The colors of the phylogenetic tree correspond to the annotations on the right side

**Fig. 2:**
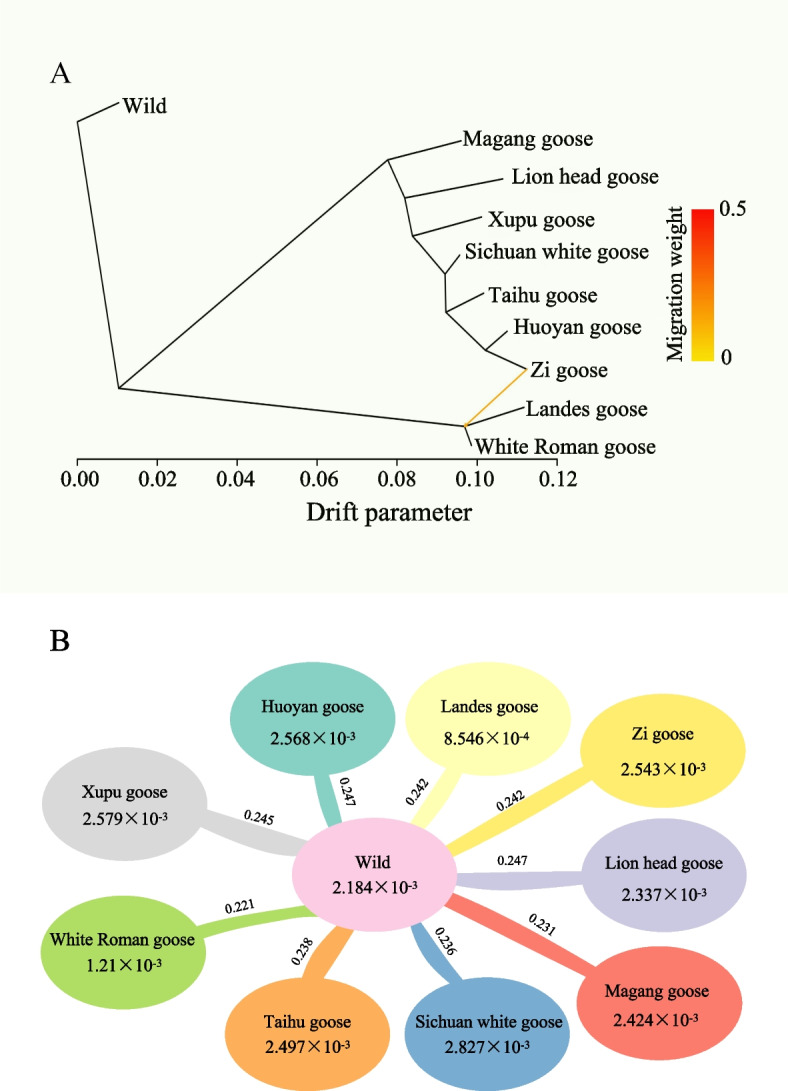
Analysis of gene flow and genetic diversity across diverse populations of geese. **A** Analysis of gene flow between various populations of geese. Colored arrows in the figure represent migration events, with the color intensity indicating the migration weight. **B** The nucleotide diversity (*π*) values of wild geese and various domestic goose breeds were calculated, along with their fixation index (*F*st) compared with wild geese. The numbers inside the ellipses represent the *π* value, and the values on the lines between the ellipses indicate the fixation index

In this study, we generated 378 WGS datasets of 9 Chinese or European domestic geese breeds with sequencing depths ranging from 8.95× to 43.24× , representing 9 breeds cultivated in diverse regions (Fig. 1A). By employing the BWA-GATK SNP calling pipeline, we detected a total of 10,072,006 high-quality SNPs. Based on the SNP data, the analysis of population structure demonstrated the most pronounced concordance between the ADMIXTURE analysis and the phylogenetic tree at K = 8. The phylogenetic tree reveals that geographically adjacent breeds share close genetic affinities, as exemplified by the Huoyan goose and Zi goose, as well as the Xupu goose and Lion head goose (Fig. 1A and B). However, a noteworthy distinction emerges in the 1-year-old female Sichuan white goose population compared to the 70-day-old male Sichuan white goose population (Fig. 1B), which could be attributed to the influence of sex chromosome sequence. Additionally, the gene flow analysis conducted among distinct breeds, including Zi goose and White Roman goose, unveiled the evidence of genetic interchange (Fig. 2A), suggesting the potential occurrence of hybridization among these distinct breeds.

To investigate the process of domestication and trait improvement in geese, we examined the nucleotide diversity within domesticated populations resulting from the selective pressures of specific traits. The analysis of nucleotide diversity (*π*) indicated that Landes and White Roman geese displayed the lowest *π* values (Fig. 2B). Whereas, the Sichuan white geese exhibited the highest *π* value (2.827 × 10^–3^), which may be related to the hybridization with other breeds. Comparative analysis of *F*st values across the entire genome revealed marked genetic differentiation between all domesticated goose breeds and all the wild geese (0.2207 to 0.2467), which was higher than the genetic differentiation observed between domesticated goose and wild populations.

### Artificial selection pressure shaping the economic traits of domestic geese

**Fig. 3:**
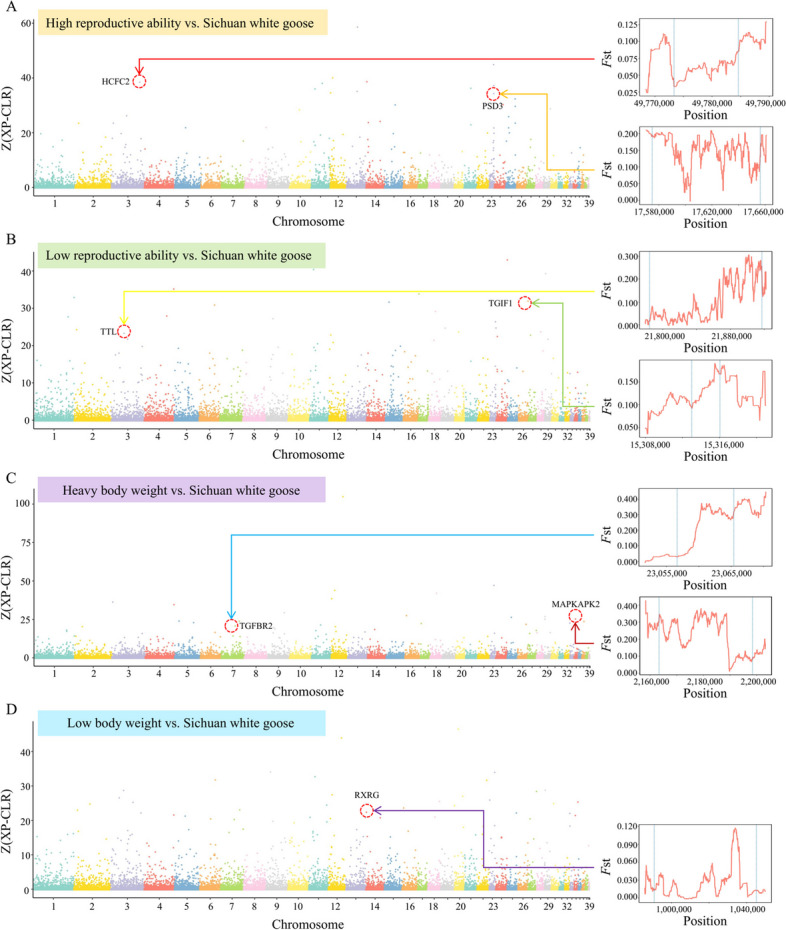
The analysis of selection signals during the domestication process of geese involving populations of geese with various characteristics and interpopulation selection signal analyses. **A** Geese with high reproductive ability versus Sichuan white geese; to identify the selection signals between goose breeds with high reproductive ability and Sichuan white geese. **B** Geese with low reproductive ability versus Sichuan white geese; to identify the selection signals between goose breeds with low reproductive ability and Sichuan white geese. Sichuan white geese are medium-weight geese. **C** Geese with heavy body weight versus Sichuan white geese; to identify the selection signals between breeds of high body weight and Sichuan white geese. **D** Geese with low body weight versus Sichuan white geese; to identify the selection signals between breeds of low body weight and Sichuan white geese. *HCFC2* Host cell factor C2, *PSD3* Pleckstrin and Sec7 domain containing 3, *TGIF1* TGFB-induced factor homeobox 1, *TTL* Tubulin-tyrosine ligase family, *TGFBR2* Transforming growth factor beta receptor 2, *MAPKAPK2* Mitogen-activated protein kinase-activated protein kinase 2, *RXRG* Retinoid X receptor gamma. The breeds with high body weight include Lion head goose. The breeds with low body weight include Huoyan goose, Zi goose, Magang goose, and Taihu goose. The breeds with high-reproductive-ability include Huoyan goose and Zi goose, while the breeds with low-reproductive-ability include Xupu goose, Magang goose, and Lion head goose

To explore the genetic foundations of goose reproduction and body weight traits, we conducted a cross-population analysis involving Sichuan white geese (exhibiting moderate reproductive ability and medium body weight) and other breeds, including those with high reproductive ability (Huoyan and Zi goose), low reproductive ability (Xupu, Magang, and Lion head goose), heavy body weight (Lion head goose), and low body weight (Huoyan, Zi, Magang goose, and Taihu goose). For detail, we utilized the cross-population composite likelihood ratio (XP-CLR) and *F*st method to explore variations in allele frequencies among a range of domesticated goose breeds that display distinct characteristics. The XP-CLR and *F*st analysis was conducted on the population with high reproductive ability geese breeds and Sichuan white geese. A total of 4,093 strong selection signal regions were identified, covering 79.31 Mb and containing 2,311 genes between populations with strong reproductive ability and Sichuan white geese (Fig. 3A, Table S3). Through *F*st calculations, significant genetic differences were observed between populations with high reproductive ability breeds and Sichuan white geese, particularly in the genes *HCFC2* on chromosome 3 and *PSD3* on chromosome 23. The XP-CLR and *F*st analysis was conducted on the population with the low reproductive ability breeds and Sichuan white geese; strong selection signal regions of 80.03 Mb containing 2,370 genes were identified (Fig. 3B). The upstream 4 kb and first half of the tubulin-tyrosine ligase family gene exhibited little genetic difference between the populations with low reproductive ability and Sichuan white geese; however, the second half of the gene and downstream 4 kb exhibited significant genetic differences. In this study, the XP-CLR analysis was conducted using Sichuan white goose to identify selection signals related to body weight via comparison with large- and small-body weight geese. The strong selection signal region between Sichuan white goose and breeds with heavy and low body weight was 81.97 Mb (Fig. 3C) and 80.08 Mb (Fig. 3D), respectively.

### SNP-GWAS and haplotype analysis for the economic traits

**Fig. 4:**
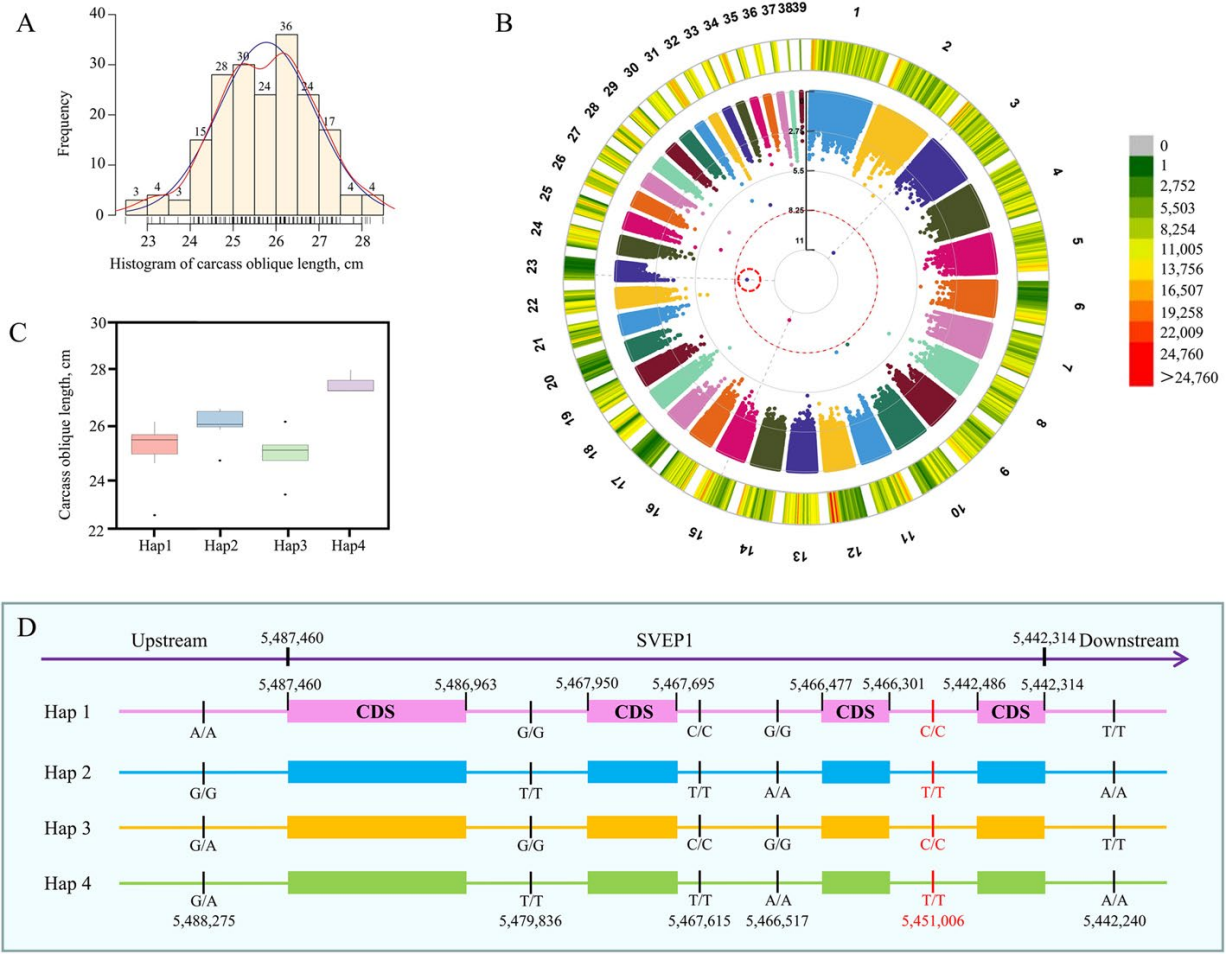
GWAS and haplotype analysis for carcass oblique length in geese. **A** Frequency distribution of carcass oblique length in 209 Sichuan white geese. **B** Manhattan plot of GWAS results for carcass oblique length in Sichuan white geese with SNP markers; the threshold is 5.63 × 10^−9^. The color scale (green to red) represents the density of SNPs in various regions of the genome. **C** Phenotypic distribution of various haplotypes of *SVEP1*, which was identified by GWAS to be associated with the carcass oblique length. **C** Frequency distribution of carcass oblique length in 209 Sichuan white geese. **D** SNP distribution of four different haplotypes of *SVEP1*, including the gene region and 1-kb upstream and 500-bp downstream of the gene. The SNP in red font is located within the intronic region of the gene

To gain further insight into the impact of artificial selection on goose traits, this study conducted GWAS to analyze critical traits, such as 70-day body weight, egg index, fertility, etc. Specifically, body weight was identified as an essential breeding trait for geese, and these traits were examined in a cohort of 432 geese in this investigation (Table S4). Employing a significance threshold of 5.63 × 10^–9^, the GWAS study uncovered a significant or highly significant correlation between 44 SNPs and seven phenotypic traits (Fig. S1, Table S5). A total of 203 Sichuan male white geese at the age of 70 d were examined to measure their oblique carcass length, which ranged from 22.5 to 28.5 cm (Fig. 4A). The GWAS analysis of this trait identified three SNP loci (chr3:6,498,019, chr15:16,559,494 and chr23:5,451,006) significantly associated with the phenotype (Fig. 4B).

Linkage disequilibrium (LD) regions indicate that significant SNP sites may not necessarily be causal variants. Analyzing the relationship between haplotypes (combinations of SNPs in a gene or promoter region) and phenotypes associated with GWAS signals is a good way to determine the relationship between the variation and phenotype. Using pentraxin domain-containing protein 1 (*SVEP1*) (at position 5,442,314–5,487,460 on chromosome 23) associated with carcass oblique length as an example, 287 nucleotide polymorphism sites were identified in the CDS region, 1-kb upstream and 500-bp downstream of this gene. Based on these sites, 182 haplotypes were identified in 209 Sichuan white geese, with Hap1 being the most common (10 samples). The sample with the most extended carcass oblique length had Hap4 as the haplotype for *SVEP* (Fig. 4C). It is worth noting that the difference between Hap4 and Hap2 of *SVEP* is only in the upstream region (at position 5,488,275 on chromosome 23) of the gene (Fig. 4D), indicating that variations in the gene’s *cis*-regulatory region may affect the phenotype by regulating gene expression.

### Construction of goose pan-genome

**Fig. 5:**
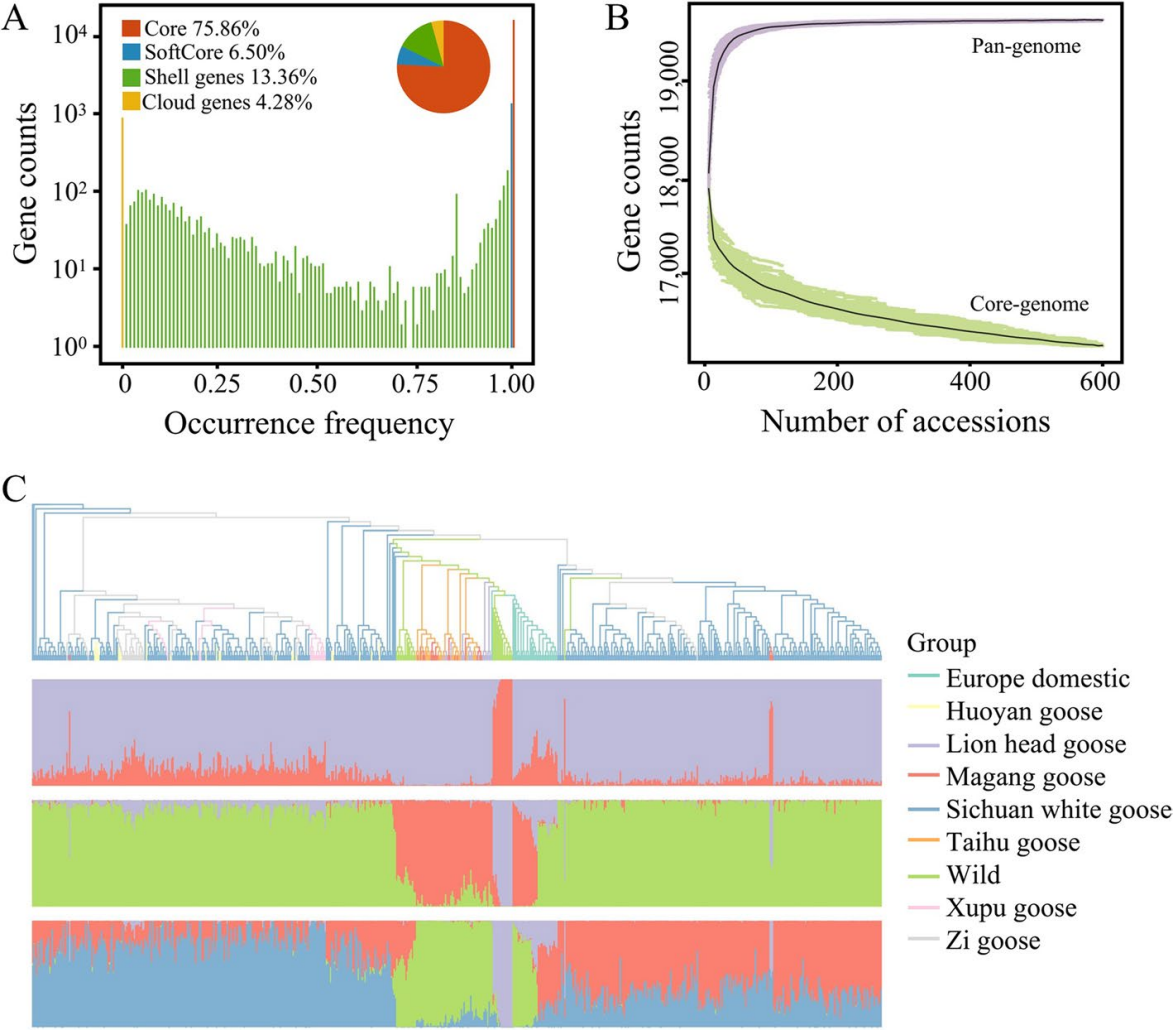
The construction of the goose pan-genome and the gene PAV analysis of the population. **A** The distribution of the number of core, softcore, shell, and cloud genes. **B** Simulation of the core gene number and total number of genes in the pan-genome was performed as a function of increasing sample size using the pan-genome constructed from the resequencing data of all geese as a reference. The process was repeated 100 times by randomly subsampling each sample size. **C** The phylogenetic tree and population structure of geese based on binary gene PAV data

To investigate genomic sequences beyond the single reference genome sequence, we conducted pan-genome estimation for gene PAV detection construction analysis by comparing various genome versions (Tianfu goose, Sichuan white goose, and Zhedong while goose). Despite limitations in the assembly of contigs using second-generation sequencing data, including shorter contig lengths, this strategy still proved valuable in obtaining 612 Mb of a new sequence, 2,813 new genes, and a total of 20,503 genes across the pan-genome. Mapping the WGS data to the pan-genome allowed the identification of gene PAV, with the identification of 15,567 core genes, 1,324 softcore genes, 2,734 shell genes, and 878 cloud genes (Fig. 5A). Core genes refer to the set of genes present in all accessions, while softcore genes are those found in 99% to 100% of accessions. Shell genes encompass the group of genes present in 1% to 99% of accessions, whereas cloud genes are defined as those with occurrence in less than 1% of accessions. The core genes comprised 75.9% of the total genes of geese; based on data simulations, as the goose population size reached 100, the pan-genome tended to saturation, while the number of core genes exhibited a consistent decline (Fig. 5B).

The phylogenetic tree constructed based on gene PAV revealed population relationships and structures that differed from the tree constructed based on SNPs (Fig. 5C). For example, the Sichuan white goose population shows almost no admixture with other breeds in the SNP-based phylogenetic tree. However, in the gene PAV-based phylogenetic tree, some breeds, such as the Magang goose, Huoyan goose, and Zi Goose, exhibit admixture with the Sichuan white goose population. Furthermore, the wild species are clustered closely together on the phylogenetic tree (Fig. 1B), indicating the absence of significant hybridization events. However, the phylogenetic tree constructed based on gene PAV suggests that there might be certain hybridization events between wild and domesticated geese. This provided further insights into geese breeds’ genetic relatedness and hybridization processes. Due to the hybridization of two breeds, their unique genes are inherited jointly by the offspring, which can influence gene PAV.

**Fig. 6:**
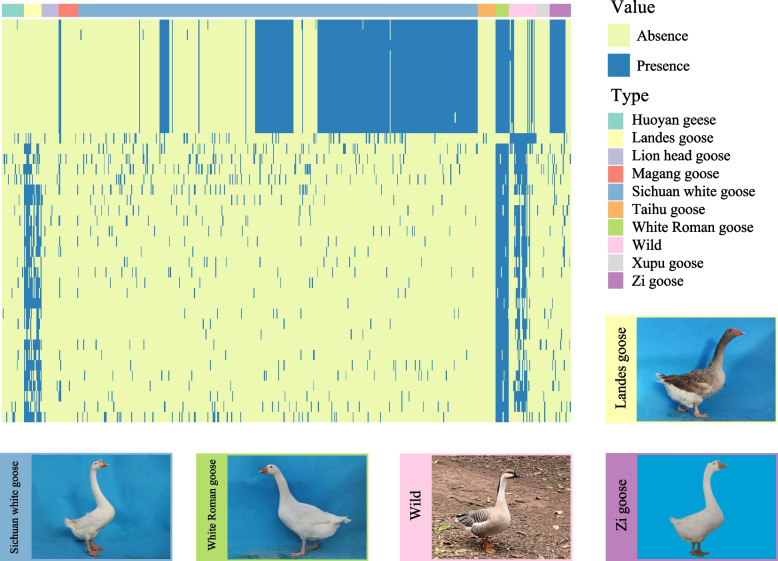
Gene PAV analysis in the goose pan-genome. The PAV heatmap of shell genes, revealing some genes specific to certain breeds. The color of the dashed box in the heatmap corresponds to the color of the box surrounding the photo of the geese

Calculating the frequency of genes in each population revealed that many breeds have unique genes (Fig. 6). All the wild goose species has the unique gene *GDPD5* (glycerophosphodiester phosphodiesterase domain-containing protein 5-like). The discovery of unique genes is vital for goose breeding and breed identification. The formation of shell genes in geese could be partly attributed to genetic differences between all the wild goose, selective breeding, and genetic drift. The functional enrichment analysis of shell genes revealed interesting associations with various economic traits of geese (Fig. S2, Table S6), such as “hair follicle maturation”, which is involved in the complex and long-term physiological process of regulating the growth and development of feathers. Since feathers and down are important products of the light industry in geese, the abundance of PAV in genes associated with “hair follicle maturation” GO term may be related to the differences in feather characteristics among various geese breeds.

### Selection of gene PAVs and gene PAV-based GWAS in Sichuan white geese

**Fig. 7:**
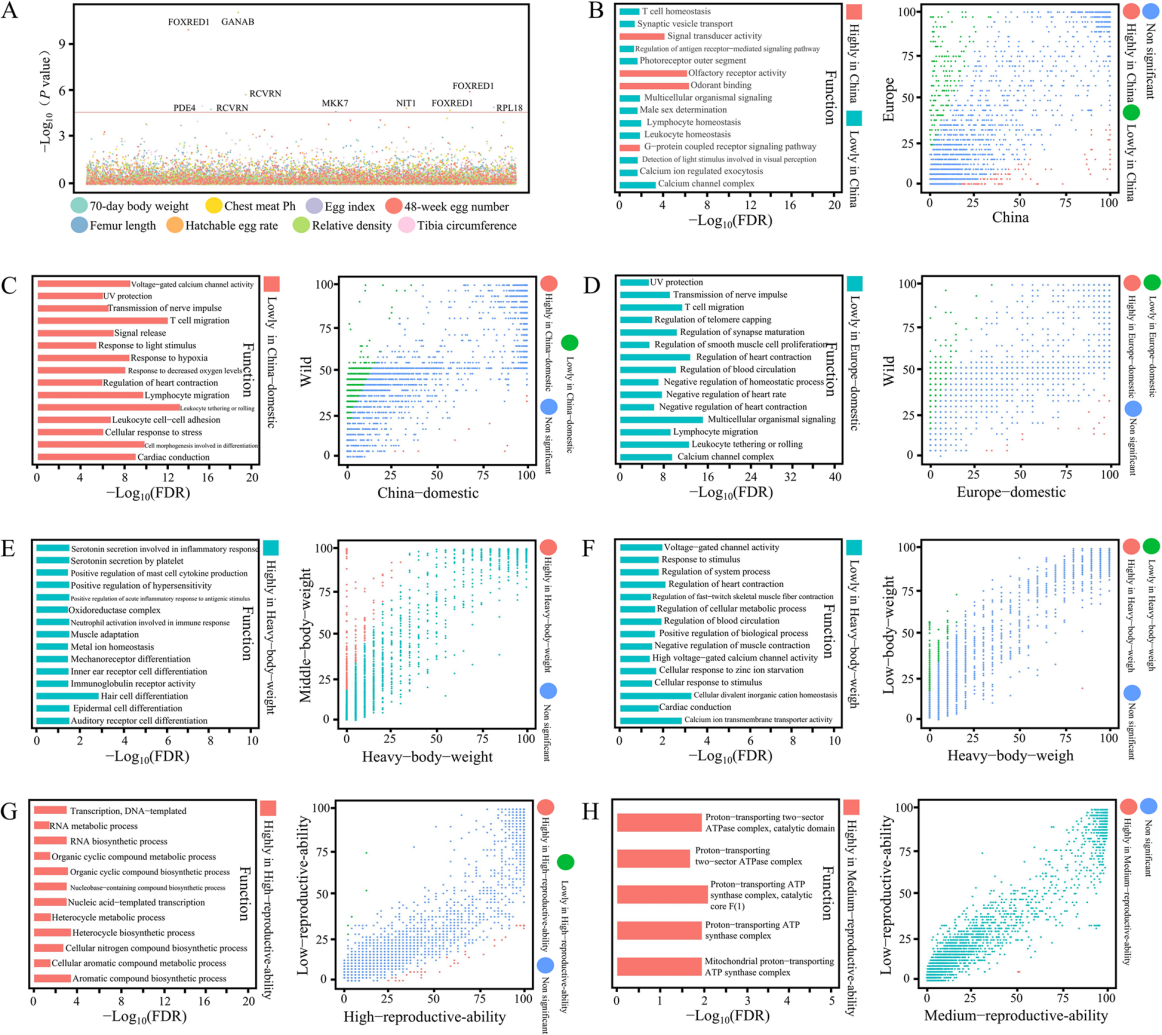
PAV-GWAS and gene frequencies between geese breeds. **A** Manhattan plot of gene PAV-GWAS for shell genes located outside the reference genome. The analysis was conducted for eight different phenotypes, and significantly associated genes were labeled with the abbreviations of proteins as follows: RCVRN: Recoverin, FOXRED1: FAD-dependent oxidoreductase domain-containing protein 1, RPL18: Large subunit ribosomal protein L18e, MKK7: Mitogen-activated protein kinase kinase 7, NIT1: Nitrilase homolog 1, GANAB: Mannosyl-oligosaccharide alpha-1,3-glucosidase, and PDE4: cAMP-specific phosphodiesterase 4. **B** Comparison analysis of gene frequencies between domestic geese in China and domestic geese in Europe, as well as GO enrichment analysis results of genes with frequency differences. The domestic geese from China include Sichuan white goose, Huoyan goose, Xupu goose, Zi goose, Magang goose, Lion head goose, and Taihu goose, while the domestic geese from Europe include White Roman goose and Landes goose. **C** Comparison analysis of gene frequencies between domestic geese in China and wild geese, as well as GO enrichment analysis results of genes with frequency differences. The wild geese species include Bar-headed goose, Barnacle goose, Black Brent goose, Cackling goose, Dark-bellied Brent goose, Emperor goose, Greater White-fronted goose, Greylag goose, Lesser White-fronted goose, Pink-footed goose, Red-breasted goose, Wan goose, Taiga Bean goose, and Tundra Bean goose. **D** Comparison analysis of gene frequencies between domestic geese in Europe and wild geese, as well as GO enrichment analysis results of genes with frequency differences. **E** Comparison analysis of gene frequencies between domestic geese with high body weight and medium body weight, as well as GO enrichment analysis results of genes with frequency differences. The breeds with medium body weight include Sichuan white goose and Xupu goose. **F** Comparison analysis of gene frequencies between domestic geese with high body weight and low body weight, as well as GO enrichment analysis results of genes with frequency differences. **G** Comparison analysis of gene frequencies between domestic geese with high-reproductive-ability and low-reproductive-ability, as well as GO enrichment analysis results of genes with frequency differences. **H** Comparison analysis of gene frequencies between domestic geese with medium-reproductive-ability and low-reproductive-ability, as well as GO enrichment analysis results of genes with frequency differences. The breeds with medium-reproductive-ability include Taihu goose and Sichuan white goose. The threshold for significantly different gene frequencies was set at a fold change > 2 and *P*_adj_ < 0.001

Humans’ domestication process of geese can be analyzed through SNP analysis and gene PAV analysis, revealing novel insights into genomic selective pressures. Using the binary PAV of shell genes as genotypes, GWAS was performed with various phenotypes, including 70-day body weight, chest meat pH, egg index, egg number at 48 weeks, femur length, fertility, egg relative density, and tibia circumference in geese. In this study, we identified 1,906 shell genes as non-reference novel genes. Among them, ten novel genes (e.g., *FOXRED1*, *GANAB*, and *RCVRN*) were found to be associated with these phenotypes (Fig. 7A, Table S7). Two genes were associated with egg number at 48 weeks and egg relative density-the association of these novel gene PAVs with phenotypes allowed identifying new candidate genes.

The animal breeding process involves hybridization, selection, and other factors that can cause changes in gene frequency across populations. Comparing gene frequency between populations, particularly those with different phenotypic characteristics, can help identify gene PAVs under selection (Table S8). The ancestral lineages of geese between Central Europe and China are different, which may result in genetic background differences between these two populations. Comparative analysis revealed 118 high- and 167 low-frequency genes in Chinese geese (Fig. 7B). In Chinese geese, 17 genes have a higher frequency than in European geese. Compared with wild geese, 696 low-frequency genes and only 13 high-frequency genes are present in Chinese indigenous geese (Fig. 7C). This indicates that some genes were lost during domestication and breeding, which may have participated in the regulation of important traits. For example, 33 genes belonging to the GO term “regulation of heart contraction” in the pan-genome are low-frequency genes in Chinese indigenous goose (out of 25 genes). Similarly, European domesticated geese have lost many genes (19 high- and 662 low-frequency genes) compared with the wild geese (Fig. 7D). The GO terms enriched in the low-frequency genes in European geese were similar to those in Chinese geese, such as “regulation of heart contraction” and “UV protection".

Similarly, we conducted PAV-GWAS using the measured phenotypes of Sichuan white geese. The body weight of geese is an important economic trait. A comparison of the body size of geese from three groups (high body weight, medium body weight, and low body weight) revealed a significant difference in the frequency of genes. Notably, geese with high body weight had 320 high-frequency genes and only one low-frequency gene compared with the geese with medium body weight (Fig. 7E) and 338 low-frequency genes and only one high-frequency gene compared with the geese with low body weight (Fig. 7F). Genes related to muscle adaptation are affected by various drivers during evolution, such as the number of oxidative fibers in flight muscles or distance of oxygen diffusion through cells, which can affect muscle growth. Among the GO terms related to muscle adaptation, five GO terms exhibited lower frequency in geese with high body weight than those with medium body weight. This observation suggests a potential association between genes related to these five GO terms and body weight. Comparative analysis of gene frequency between geese with high and low reproductive ability (Fig. 7G) revealed that geese with high reproductive ability exhibited more high-frequency genes (24) than low-frequency genes (4). High-frequency genes in geese with high reproductive ability were enriched in many metabolic pathways, such as the “cellular aromatic compound metabolic process” and “aromatic compound biosynthetic process”, which may be involved in regulating biological activities. Analysis of gene frequency between geese with low and medium reproductive ability revealed differences in the frequency of only 11 genes, and all exhibited high frequency in geese with medium reproductive ability (Fig. 7H). This suggested that gene variants may not cause the difference between these two groups.

### Gene expression atlas of multiple organs and tissues in geese

**Fig. 8:**
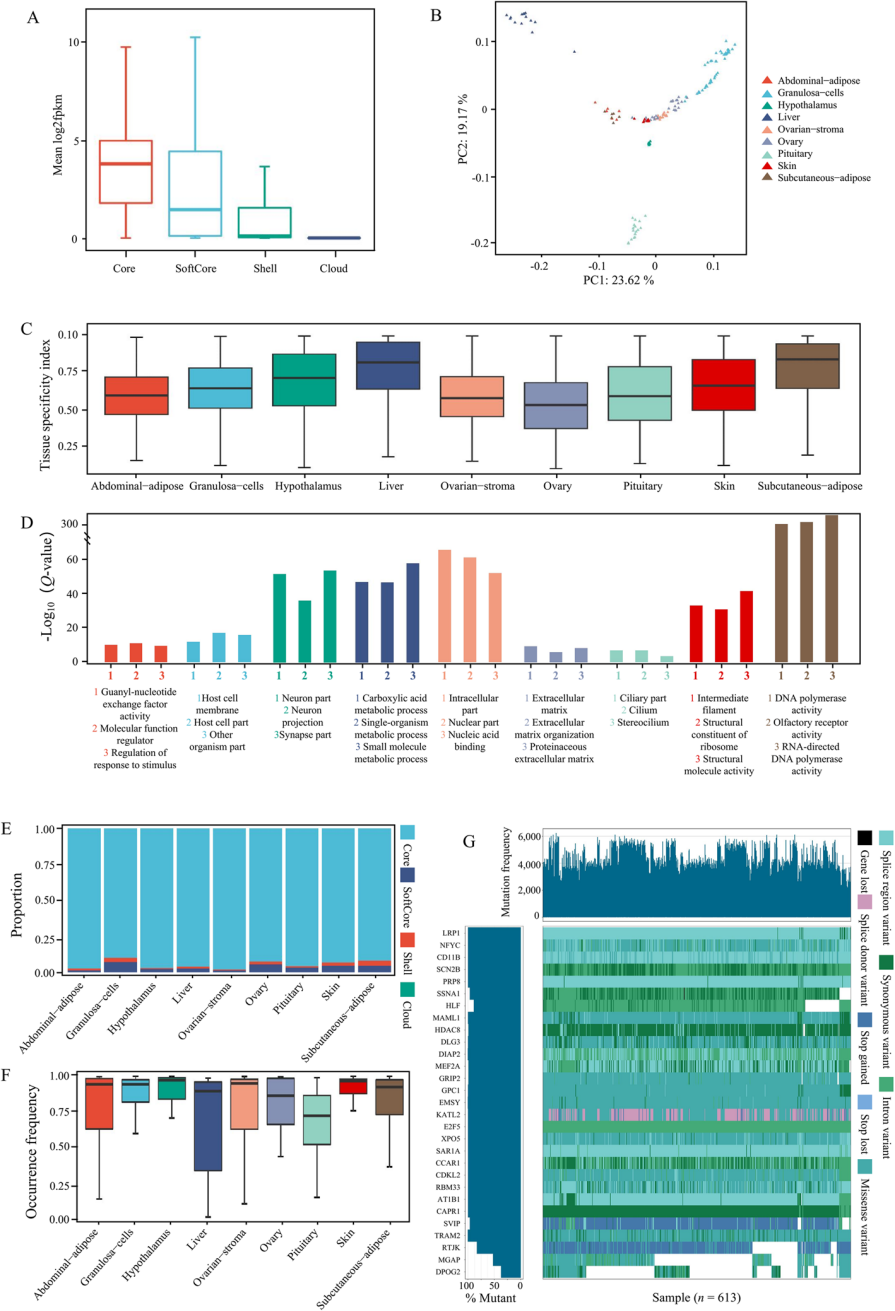
Transcriptional profiling analysis of multiple organs and tissues in geese. **A** Expression level distribution of core, softcore, shell, and cloud genes in the reference genome. **B** PCA analysis of gene expression levels in all organs and tissues. **C** Distribution of the tissue-specificity index (TAU) of gene expression in various organs and tissues. **D** GO enrichment analysis results of tissue-specific genes. **E** Proportions of tissue-specific genes classified into core, softcore, shell, and cloud genes based on the classification information of genes. **F** Gene frequency distribution of tissue-specific genes in the population of geese based on PAV information. **G** Waterfall plot of the variation burden of differentially expressed genes in the ovary during egg-production process in the population of geese. LRP1: Low-density lipoprotein receptor-related protein 1, NFYC: Nuclear transcription factor Y subunit gamma, CD11B: Cyclin-dependent kinase 11B, SCN2B: Sodium channel subunit beta-2, PRP8: Pre-mRNA-processing-splicing factor 8, SSNA1: Sjoegren syndrome nuclear autoantigen 1, HLF: Hepatic leukemia factor, MAML1: Mastermind-like protein 1, HDAC8: Histone deacetylase 8, DLG3: Disks large homolog 3, DIAP2: Protein diaphanous homolog 2, MEF2A: Myocyte-specific enhancer factor 2A homolog, GRIP2: Glutamate receptor-interacting protein 2, GPC1: Glypican-1, EMSY: BRCA2-interacting transcriptional repressor EMSY, KATL2: Katanin p60 ATPase-containing subunit A-like 2, E2F5: Transcription factor E2F5, XPO5: Exportin-5, SAR1A: GTP-binding protein SAR1a, CCAR1: Cell division cycle and apoptosis regulator protein 1, CDKL2: Cyclin-dependent kinase-like 2, RBM33: RNA-binding protein 33, AT1B1: Sodium/potassium-transporting ATPase subunit beta-1, CAPR1: Caprin-1, SVIP: Small VCP/p97-interacting protein, TRAM2: Translocating chain-associated membrane protein 2, RTJK: RNA-directed DNA polymerase from mobile element jockey, MGAP: MAX gene-associated protein, and DPOG2: DNA polymerase subunit gamma-2, mitochondrial

While previous studies have revealed various variations and genes associated with domestication and selection through population genomics and pan-genomics analyses, a more comprehensive understanding of gene functions has been limited due to the need for more integration with gene expression information. Transcriptome analysis is essential for studying animal growth, development, and environmental adaptation. Therefore, this study aims to advance the investigation of the goose genome by conducting a large-scale integrated analysis of transcriptomic data. We downloaded transcriptomes from 9 organs and tissues of geese, including abdominal adipose tissue, granulosa cells, hypothalamus, liver, ovarian stroma, ovary, pituitary, skin, and subcutaneous adipose tissue (Table S9). The core genes exhibited higher expression levels in all samples. In contrast, low-frequency genes exhibited lower expression levels (Fig. 8A). Highly conserved genes play fundamental and essential roles in various life activities of geese. Although the expression levels of cloud genes were low, their importance should not be ignored as they may be related to various phenotypes in various goose breeds. PCA analysis revealed the specificity of gene expression in various organs and tissues of geese (Fig. 8B). Genes with organ specificity may be related to various functions of different organs of geese. TAU value calculation revealed that all organs and tissues had genes with specific expression levels (Table S10). The liver and ovary had the highest and lowest specificity index, respectively, for specifically expressed genes (Fig. 8C). In the hypothalamus, these specifically expressed genes were enriched in some specific functions, such as synapse part, neuron part, and neuron projection (Fig. 8D, Table S11). Gene PAV analysis revealed that although the core specifically expressed genes had the highest proportion in all organs of geese, the proportion of variable genes differed in various organs. The proportion of variable genes was the highest and lowest in subcutaneous adipose tissue (7.9%) and abdominal adipose tissue (2.5%), respectively (Fig. 8E). However, the distribution of gene frequency did not correspond to the proportion of variable genes (Fig. 8F). For example, the specifically expressed genes in the liver had the highest median gene frequency; however, they did not have the lowest proportion of variable genes.

Reproductive ability is an essential indicator for measuring goose breeds’ production capacity and economic benefits, which is significant for the breeding and animal husbandry industry. This study analyzed the ovaries’ temporal differential expression during various egg-laying periods, and 30 differentially expressed genes were identified. These differentially expressed genes’ mutation load was analyzed, revealing a diverse range of mutation loads and types among various genes (Fig. 8G, Table S12). The DNA polymerase subunit gamma-2 (*DPOG2*) gene exhibited low variability in the population, and its primary type of mutation was the intron variant, indicating that it was unlikely to be involved in shaping the reproductive ability trait in geese. Meanwhile, other genes, such as Sjoegren syndrome nuclear autoantigen 1 (*SSNA1*), underwent gene loss in one Lion head goose. Notably, many splice region variants were discovered in the gene region of low-density lipoprotein receptor-related protein 1 (*LRP1*). These mutations primarily caused gene splicing, affecting the protein product and leading to further phenotype changes. In summary, these results enhanced the understanding of the variation of these genes in the goose population.

## Discussion

### Genetic diversity and gene flow in goose population

In this study, our findings are in accordance with previous research, supporting the domestication of geese from either the Swan or Greylag goose [3, 4, 72]. Furthermore, the gene flow analysis presents compelling evidence of genetic exchange between the Zi goose and the shared ancestor of the Landes and White Roman goose breeds (Fig. 2A). This suggests the possibility of hybridization among these distinct breeds, akin to the hybridization observed in species mated in their natural environments and captivity [4, 18, 32, 73], which consistent the previous study [18]. A comparison analysis of *F*st values across the whole genome revealed that significant genetic differentiation existed between all domesticated goose breeds and wild geese (0.2207 to 0.2467), which was higher than the genetic differentiation observed between domesticated and wild populations of other poultry species, such as chicken [74].

### Genomic selection regions for reproduction and body weight traits

The analysis of Chinese indigenous goose populations with varying reproductive abilities or body weight traits revealed that genomic regions under selection were associated with reproductive capability (*HCFC2*, *PSD3*, *TGIF1*, and *TTL*), as well as body weight (*TGFBR2*, *MAPKAPK2*, and *RXRG*). The *TGIF1* gene serves as a transcription factor, repressing TGF-β signaling and exerting a substantial influence on various processes, including embryonic development, mammalian reproduction, adipocyte differentiation, vascularization, and embryonic and gonadal development [75]. Additionally, *TGIF1* polymorphisms have been linked to litter size in sheep [76]. Furthermore, there are indications of an interaction between *TGIF1* and *SMAD2*, a well-established participant in reproductive processes [75]. The *TTL* gene encodes a vital cytosolic enzyme responsible for catalyzing the post-translational retyrosination of detyrosinated α-tubulin, a process critical to cell and organism development [77]. Previous studies have established connections between this gene and factors such as the weight of the egg at first oviposition, eggshell color, and egg quality [78–80], underscoring the need for additional inquiries to enhance our comprehension of how this gene impacts the egg-laying process in geese. For the body weight trait, the candidate gene *MAPKAPK2* (Fig. 3C), identified through XP-CLR within various body weight selection signal regions, demonstrates a strong association with chicken enteritis induced by *Salmonella* [81] and is also implicated in cell proliferation [82]. This suggested that this gene may have diverse roles in regulating geese’s growth and immune processes. This observation suggests a potential multifaceted role for this gene in governing the growth and immune processes of geese. Similarly, transforming growth factor beta receptor 2 (*TGFBR2*), a vital initiator of the TGF-β signaling pathway that oversees cell growth and organ development [83], resides within the selection signal region distinguishing breeds with high body weight from Sichuan white geese. In addition, *SVEP1* obtained from GWAS based on the phenotype of carcass oblique length is mentioned in many reports related to human tumor research and is related to the immune process [84, 85]. Further studies are needed to elucidate how this gene functions in geese and its impact on carcass oblique length.

### Missing sequence in goose genome

Interesting, our investigation revealed 612 Mb of previously undiscovered sequence within the goose genome, accomplished through a comprehensive pan-genome analysis that integrated various reference goose genomes (Tianfu goose, Sichuan white goose, and Zhedong white goose), while the total goose genome size being 1.1 to 1.2 Gb. However, in avian species with genomes of similar sizes, such as chickens and ducks, 159 Mb and 33 Mb of missing sequences were identified, respectively [86, 87]. Several studies across multiple species have demonstrated that long-term domestication can lead to differences in the number of genes among various populations [88, 89]. Goose has a domestication history of 7,000 years, with European and Chinese geese having different ancestors, abundant variation among different breeds, which could potentially explain why geese, in contrast to other monophyletic domesticated poultry, demonstrate a higher prevalence of missing sequences. In our pan-genome analysis, we found over 3,000 missing genes out of a total of 20,503 genes when compared to a single goose genome (Tianfu goose, Sichuan white goose, or Zhedong white goose), consistent with similar findings in pan-genome analyses of chickens and ducks, and these missing genes demonstrated tissue-specific expression patterns [86, 87].

## Conclusions

In conclusion, our study offers a comprehensive grasp of the domestication and breeding evolution of geese. It delves into crucial trait-associated genetic loci and sheds light on physiological attributes through sequencing analyses across diverse goose breeds. This investigation encompasses pan-genome construction, PAV analysis, and RNA expression profiling. These insights establish a robust groundwork for advancing goose research and breeding endeavors.

### Supplementary Information

**Additional file 1: Fig. S1.** The Manhattan plots of various phenotypes of Sichuan white geese. A-G represents the phenotypes of Sichuan white geese as follows: 9-20 wk geese feed to meat ratio, carcass keel bone length, chest meat pH, keel bone length, leg circumference, feet weight, and tibia length.

**Additional file 2: Fig. S2.** GO enrichment analysis results of the shell genes. The GO terms were related to biological process (A), cellular component (B), and molecular function (C).

**Additional file 3: Fig. S3.** Summary of pan-genome and transcriptome studies in the present study.

**Additional file 4: Table S1.** Samples newly sequenced in this study.

**Additional file 5: Table S2.** Resequencing data downloaded in this study.

**Additional file 6: Table S3.** Putative domestication selected regions.

**Additional file 7: Table S4.** Phenotypic data for traits of Sichuan white geese for the resequenced accessions.

**Additional file 8: Table S5.** Signals in the SNP-GWAS analysis.

**Additional file 9: Table S6.** Shell gene GO enrichment analysis.

**Additional file 10: Table S7.** Signals in the PAV-GWAS analysis.

**Additional file 11: Table S8.** Genes detected with reliable PAV calling.

**Additional file 12: Table S9.** Gene FPKM data.

**Additional file 13: Table S10.** Tissue specificity index of different organs.

**Additional file 14: Table S11.** GO enrichment analysis of organ-specific gene expression.

**Additional file 15: Table S12.** Gene variant information in the waterfall.

## Data Availability

All the WGS are publicly available through the NCBI Bioproject PRJNA595357 and PRJNA695024.

## Abbreviations

*F*st: Fixation index
GO: Gene Ontology
GWAS: Genome-wide association studies
PAV: Presence-absence variation
PCA: Principal component analysis
SNPs: Single nucleotide polymorphisms
SVEP1: Pentraxin domain-containing protein 1
TAU: The tissue specificity index
TGIF1: TGFB-induced factor homeobox 1
WGS: Whole-genome sequencing

## References

[1] Shi XW, Wang JW, Zeng FT, Qiu XP (2006). Mitochondrial DNA cleavage patterns distinguish independent origin of Chinese domestic geese and Western domestic geese. Biochem Genet.

[2] Eda M, Itahashi Y, Kikuchi H, Sun G, Hsu K-H, Gakuhari T (2022). Multiple lines of evidence of early goose domestication in a 7,000-y-old rice cultivation village in the lower Yangtze River, China. Proc Natl Acad Sci U S A.

[3] Li HF, Zhu WQ, Chen KW, Xu WJ, Song W, H Y (2011). Two maternal origins of Chinese domestic goose. Poult Sci.

[4] Wen J, Li H, Wang H, Yu J, Zhu T, Zhang J (2023). Origins, timing and introgression of domestic geese revealed by whole genome data. J Anim Sci Biotechnol.

[5] Boz MA, Sarica M, Yamak US (2017). Production traits of artificially and naturally hatched geese in intensive and free-range systems: I. Growth traits Br Poult Sci.

[6] Kozák J (2019). Variations of geese under domestication. Worlds Poult Sci J.

[7] Zhao Q, Lin Z, Chen J, Xie Z, Wang J, Feng K (2023). Chromosome-level genome assembly of goose provides insight into the adaptation and growth of local goose breeds. GigaScience.

[8] Lu L, Chen Y, Wang Z, Li X, Chen W, Tao Z, et al. The goose genome sequence leads to insights into the evolution of waterfowl and susceptibility to fatty liver. Genome Biol. 2015;16:89. 10.1186/s13059-015-0652-y. 10.1186/s13059-015-0652-y PMC4419397 25943208

[9] Gao GL, Zhao XZ, Li Q, He C, Zhao WJ, Liu SY (2016). Genome and metagenome analyses reveal adaptive evolution of the host and interaction with the gut microbiota in the goose. Sci Rep.

[10] Zhang YW, Zhang B, Zhang Y, Nie RX, Zhang J, Shang P (2022). Chromosome-level genome assembly of the bar-headed goose (Anser indicus). Sci Data.

[11] Ouyang J, Zheng SM, Huang M, Tang HB, Qiu XH, Chen SJ (2022). Chromosome-level genome and population genomics reveal evolutionary characteristics and conservation status of Chinese indigenous geese. Commun Biol.

[12] Zhang YH, Ni HY, Xie HL, Yin YJ, Zheng JL, Dong LP (2022). De novo assembly of a wild swan goose (Anser cygnoides) genome. Anim Genet.

[13] Li Y, Gao GL, Lin Y, Hu SL, Luo Y, Wang GS (2020). Pacific Biosciences assembly with Hi-C mapping generates an improved, chromosome-level goose genome. GigaScience.

[14] Karawita AC, Cheng Y, Chew KY, Challagulla A, Kraus R, Mueller RC (2023). The swan genome and transcriptome, it is not all black and white. Genome Biol.

[15] Xi Y, Wang L, Liu HH, Ma SC, Li YY, Li L, et al. A 14-bp insertion in endothelin receptor B-like (EDNRB2) is associated with white plumage in Chinese geese. BMC Genomics. 2020;21:162. 10.1186/s12864-020-6562-8. 10.1186/s12864-020-6562-8 PMC7027040 32066369

[16] Gao GL, Gao DF, Zhao XZ, Xu SS, Zhang KS, Wu R (2021). Genome-wide association study-based identification of SNPs and haplotypes associated with goose reproductive performance and egg quality. Front Genet.

[17] Zhao Q, Chen JP, Zhang XH, Xu ZY, Lin ZP, Li HX (2020). Genome-wide association analysis reveals key genes responsible for egg production of lion head goose. Front Genet.

[18] Heikkinen ME, Ruokonen M, White TA, Alexander MM, Gündüz İ, Dobney KM (2020). Long-term reciprocal gene flow in wild and domestic geese reveals complex domestication history. G3 (Bethesda).

[19] Deng Y, Hu SQ, Luo CL, Ouyang QY, Li L, Ma JM (2021). Integrative analysis of histomorphology, transcriptome and whole genome resequencing identified DIO2 gene as a crucial gene for the protuberant knob located on forehead in geese. BMC Genomics.

[20] Wen J, Shao P, Chen Y, Wang L, Lv X, Yang W (2021). Genomic scan revealed KIT gene underlying white/gray plumage color in Chinese domestic geese. Anim Genet.

[21] Zheng S, Ouyang J, Liu S, Tang H, Xiong Y, Yan X (2023). Genomic signatures reveal selection in Lingxian white goose. Poult Sci.

[22] Chen J, Zhang S, Chen G, Deng X, Zhang D, Wen H (2022). Transcriptome sequencing reveals pathways related to proliferation and differentiation of shitou goose myoblasts. Animals (Basel).

[23] Hu M, Jin H, Wu J, Zhou X, Yang S, Zhao A (2022). Identification of the differentially expressed genes in the leg muscles of Zhedong white geese (Anser cygnoides) reared under different photoperiods. Poult Sci.

[24] Ouyang Q, Hu S, Wang G, Hu J, Zhang J, Li L (2020). Comparative transcriptome analysis suggests key roles for 5-hydroxytryptamlne receptors in control of goose egg production. Genes (Basel).

[25] Gong Y, Li Y, Liu X, Ma Y, Jiang L (2023). A review of the pangenome: how it affects our understanding of genomic variation, selection and breeding in domestic animals?. J Anim Sci Biotechnol.

[26] Talenti A, Powell J, Hemmink JD, Cook EA, Wragg D, Jayaraman S (2022). A cattle graph genome incorporating global breed diversity. Nat Commun.

[27] Li R, Gong M, Zhang X, Wang F, Liu Z, Zhang L (2023). A sheep pangenome reveals the spectrum of structural variations and their effects on tail phenotypes. Genome Res..

[28] Jiang YF, Wang S, Wang CL, Xu RH, Wang WW, Jiang Y, et al. Pangenome obtained by long-read sequencing of 11 genomes reveal hidden functional structural variants in pigs. iScience. 2023;26(3):106119. 10.1016/j.isci.2023.106119. 10.1016/j.isci.2023.106119 PMC9958381 36852268

[29] Wang K, Hu H, Tian Y, Li J, Scheben A, Zhang C (2021). The chicken pan-genome reveals gene content variation and a promoter region deletion in IGF2BP1 affecting body size. Mol Biol Evol.

[30] Tian X, Li R, Fu W, Li Y, Wang X, Li M (2020). Building a sequence map of the pig pan-genome from multiple de novo assemblies and Hi-C data. Sci China Life Sci.

[31] Zhou Y, Yang L, Han X, Han J, Hu Y, Li F (2022). Assembly of a pangenome for global cattle reveals missing sequences and novel structural variations, providing new insights into their diversity and evolutionary history. Genome Res.

[32] Ottenburghs J, Megens HJ, Kraus RHS, Van Hooft P, Van Wieren SE, Crooijmans RPMA (2017). A history of hybrids? Genomic patterns of introgression in the True Geese. BMC Evol Biol.

[33] Díez-Del-Molino D, Von Seth J, Gyllenstrand N, Widemo F, Liljebäck N, Svensson M (2020). Population genomics reveals lack of greater white-fronted introgression into the Swedish lesser white-fronted goose. Sci Rep.

[34] Ottenburghs J, Honka J, Müskens GJDM, Ellegren H (2020). Recent introgression between Taiga Bean Goose and Tundra Bean Goose results in a largely homogeneous landscape of genetic differentiation. Heredity (Edinb).

[35] Kaiqi W, Weiran H, Yang Z, Qi X, Guohong C (2021). Principal component analysis of body size, reproductive traits and ecological characteristics on Chinese indigenous Goose Breeds. J Sichuan Univ.

[36] Gao GL, Chen PP, Zhou C, Zhao XZ, Zhang KS, Wu R (2022). Genome-wide association study for reproduction-related traits in Chinese domestic goose. Br Poult Sci.

[37] Li H, Durbin R (2009). Fast and accurate short read alignment with Burrows-Wheeler transform. Bioinformatics.

[38] Danecek P, Auton A, Abecasis G, Albers CA, Banks E, Depristo MA (2011). The variant call format and VCFtools. Bioinformatics.

[39] Cingolani P, Platts A, Wang LL, Coon M, Nguyen T, Wang L, et al. A program for annotating and predicting the effects of single nucleotide polymorphisms, SnpEff: SNPs in the genome of *Drosophila melanogaster* strain w^1118^; iso-2; iso-3. Fly (Austin). 2012;6(2):80–92. 10.4161/fly.19695. 10.4161/fly.19695 PMC3679285 22728672

[40] Minh BQ, Schmidt HA, Chernomor O, Schrempf D, Woodhams MD, Von Haeseler A (2020). IQ-TREE 2: new models and efficient methods for phylogenetic inference in the genomic era. Mol Biol Evol.

[41] Alexander DH, Novembre J, Lange K (2009). Fast model-based estimation of ancestry in unrelated individuals. Genome Res.

[42] Pickrell J, Pritchard J. Inference of population splits and mixtures from genome-wide allele frequency data. Nat Prec. 2012. 10.1038/npre.2012.6956.1. 10.1371/journal.pgen.1002967 PMC3499260 23166502

[43] Cai X, Sun X, Xu C, Sun H, Wang X, Ge C (2021). Genomic analyses provide insights into spinach domestication and the genetic basis of agronomic traits. Nat Commun.

[44] Chen H, Patterson N, Reich D (2010). Population differentiation as a test for selective sweeps. Genome Res.

[45] Sherman RM, Forman J, Antonescu V, Puiu D, Daya M, Rafaels N (2019). Assembly of a pan-genome from deep sequencing of 910 humans of African descent. Nat Genet.

[46] Zimin AV, Marçais G, Puiu D, Roberts M, Salzberg SL, Yorke JA (2013). The MaSuRCA genome assembler. Bioinformatics.

[47] Marçais G, Delcher AL, Phillippy AM, Coston R, Salzberg SL, Zimin A (2018). MUMmer4: a fast and versatile genome alignment system. PLoS Comput Biol.

[48] Fu L, Niu B, Zhu Z, Wu S, Li W (2012). CD-HIT: accelerated for clustering the next-generation sequencing data. Bioinformatics.

[49] Wood DE, Lu J, Langmead B (2019). Improved metagenomic analysis with Kraken 2. Genome Biol..

[50] Tahir UI, Qamar M, Zhu X, Xing F, Chen LL (2019). ppsPCP: a plant presence/absence variants scanner and pan-genome construction pipeline. Bioinformatics.

[51] Song JM, Guan Z, Hu J, Guo C, Yang Z, Wang S (2020). Eight high-quality genomes reveal pan-genome architecture and ecotype differentiation of Brassica napus. Nat Plants.

[52] Gao GL, Hu SL, Zhang KS, Wang HW, Xie YH, Zhang CL (2021). Genome-wide gene expression profiles reveal distinct molecular characteristics of the goose granulosa cells. Front Genet.

[53] Wang G, Jin L, Li Y, Tang Q, Hu S, Xu H (2019). Transcriptomic analysis between normal and high-intake feeding geese provides insight into adipose deposition and susceptibility to fatty liver in migratory birds. BMC Genomics..

[54] Chen N (2004). Using RepeatMasker to identify repetitive elements in genomic sequences. Curr Protoc Bioinformatics.

[55] Flynn JM, Hubley R, Goubert C, Rosen J, Clark AG, Feschotte C (2020). RepeatModeler2 for automated genomic discovery of transposable element families. Proc Natl Acad Sci U S A.

[56] Benson G (1999). Tandem repeats finder: a program to analyze DNA sequences. Nucleic Acids Res.

[57] Kim D, Paggi JM, Park C, Bennett C, Salzberg SL (2019). Graph-based genome alignment and genotyping with HISAT2 and HISAT-genotype. Nat Biotechnol.

[58] Grabherr MG, Haas BJ, Yassour M, Levin JZ, Thompson DA, Amit I (2011). Trinity: reconstructing a full-length transcriptome without a genome from RNA-Seq data. Nat Biotechnol.

[59] Holt C, Yandell M (2011). MAKER2: an annotation pipeline and genome-database management tool for second-generation genome projects. BMC Bioinformatics..

[60] Stanke M, Keller O, Gunduz I, Hayes A, Waack S, Morgenstern B (2006). AUGUSTUS: ab initio prediction of alternative transcripts. Nucleic Acids Res.

[61] Langmead B, Salzberg SL (2012). Fast gapped-read alignment with Bowtie 2. Nat Methods.

[62] Golicz AA, Martinez PA, Zander M, Patel DA, Van De Wouw AP, Visendi P (2015). Gene loss in the fungal canola pathogen Leptosphaeria maculans. Funct Integr Genomics.

[63] Nguyen LT, Schmidt HA, Von Haeseler A, Minh BQ (2015). IQ-TREE: a fast and effective stochastic algorithm for estimating maximum-likelihood phylogenies. Mol Biol Evol.

[64] Hubisz MJ, Falush D, Stephens M, Pritchard JK (2009). Inferring weak population structure with the assistance of sample group information. Mol Ecol Resour.

[65] Liu X, Huang M, Fan B, Buckler ES, Zhang Z (2016). Iterative usage of fixed and random effect models for powerful and efficient genome-wide association studies. PLoS Genet.

[66] Yang J, Lee SH, Goddard ME, Visscher PM (2011). GCTA: a tool for genome-wide complex trait analysis. Am J Hum Genet.

[67] Li X, Shi Z, Gao J, Wang X, Guo K (2023). CandiHap: a haplotype analysis toolkit for natural variation study. Mol Breed.

[68] Liao Y, Smyth GK, Shi W (2014). featureCounts: an efficient general purpose program for assigning sequence reads to genomic features. Bioinformatics.

[69] Conesa A, Nueda MJ, Ferrer A, Talón M (2006). maSigPro: a method to identify significantly differential expression profiles in time-course microarray experiments. Bioinformatics.

[70] Love MI, Huber W, Anders S (2014). Moderated estimation of fold change and dispersion for RNA-seq data with DESeq2. Genome Biol.

[71] Mclaren W, Gil L, Hunt SE, Riat HS, Ritchie GR, Thormann A (2016). The ensembl variant effect predictor. Genome Biol.

[72] Heikkinen M, Ruokonen M, Alexander M, Aspi J, Pyhäjärvi T, Searle JB (2015). Relationship between wild greylag and European domestic geese based on mitochondrial DNA. Anim Genet.

[73] Ottenburghs J, Van Hooft P, Van Wieren SE, Ydenberg RC, Prins HH (2016). Hybridization in geese: a review. Front Zool.

[74] Wang MS, Zhang JJ, Guo X, Li M, Meyer R, Ashari H (2021). Large-scale genomic analysis reveals the genetic cost of chicken domestication. BMC Biol.

[75] Hu Y, Yu H, Shaw G, Renfree MB, Pask A (2011). Differential roles of TGIF family genes in mammalian reproduction. BMC Dev Biol..

[76] Zhang Z, He X, Liu Q, Tang J, Di R, Chu M (2020). TGIF1 and SF1 polymorphisms are associated with litter size in Small Tail Han sheep. Reprod Domest Anim.

[77] Prota AE, Magiera MM, Kuijpers M, Bargsten K, Frey D, Wieser M (2013). Structural basis of tubulin tyrosination by tubulin tyrosine ligase. J Cell Biol.

[78] Liu Z, Sun C, Yan Y, Li G, Shi F, Wu G (2018). Genetic variations for egg quality of chickens at late laying period revealed by genome-wide association study. Sci Rep.

[79] Gao JF, Xu W, Zeng T, Tian Y, Wu CQ, Liu SZ (2022). Genome-wide association study of egg-laying traits and egg quality in LingKun chickens. Front Vet Sci.

[80] Zhang GX, Fan QC, Wang JY, Zhang T, Xue Q, Shi HQ (2015). Genome-wide association study on reproductive traits in Jinghai Yellow Chicken. Anim Reprod Sci.

[81] Ghebremicael SB, Hasenstein JR, Lamont SJ (2008). Association of interleukin-10 cluster genes and Salmonella response in the chicken. Poult Sci.

[82] Schindler JF, Godbey A, Hood WF, Bolten SL, Broadus RM, Kasten TP (2002). Examination of the kinetic mechanism of mitogen-activated protein kinase activated protein kinase-2. Biochim Biophys Acta.

[83] Ning B, Huang J, Xu H, Lou Y, Wang W, Mu F (2022). Genomic organization, intragenic tandem duplication, and expression analysis of chicken TGFBR2 gene. Poult Sci.

[84] Michelini S, Amato B, Ricci M, Serrani R, Veselenyiova D, Kenanoglu S (2021). SVEP1 is important for morphogenesis of lymphatic system: possible implications in lymphedema. Lymphology.

[85] Jung IH, Elenbaas JS, Alisio A, Santana K, Young EP, Kang CJ (2021). SVEP1 is a human coronary artery disease locus that promotes atherosclerosis. Sci Transl Med.

[86] Li M, Sun C, Xu N, Bian P, Tian X, Wang X (2022). De novo assembly of 20 chicken genomes reveals the undetectable phenomenon for thousands of core genes on microchromosomes and subtelomeric regions. Mol Biol Evol.

[87] Zhu F, Yin ZT, Wang Z, Smith J, Zhang F, Martin F (2021). Three chromosome-level duck genome assemblies provide insights into genomic variation during domestication. Nat Commun.

[88] Liu Y, Du H, Li P, Shen Y, Peng H, Liu S (2020). Pan-genome of wild and cultivated soybeans. Cell.

[89] Qin P, Lu H, Du H, Wang H, Chen W, Chen Z (2021). Pan-genome analysis of 33 genetically diverse rice accessions reveals hidden genomic variations. Cell..

