# Supplementary figures and images for: Insights into genetic diversity and phenotypic variations in domestic geese through comprehensive population and pan-genome analysis
Source: J Anim Sci Biotechnol. 2023 Nov 24;14:150. doi: 10.1186/s40104-023-00944-y (PMC10675864; doi:10.1186/s40104-023-00944-y)

A

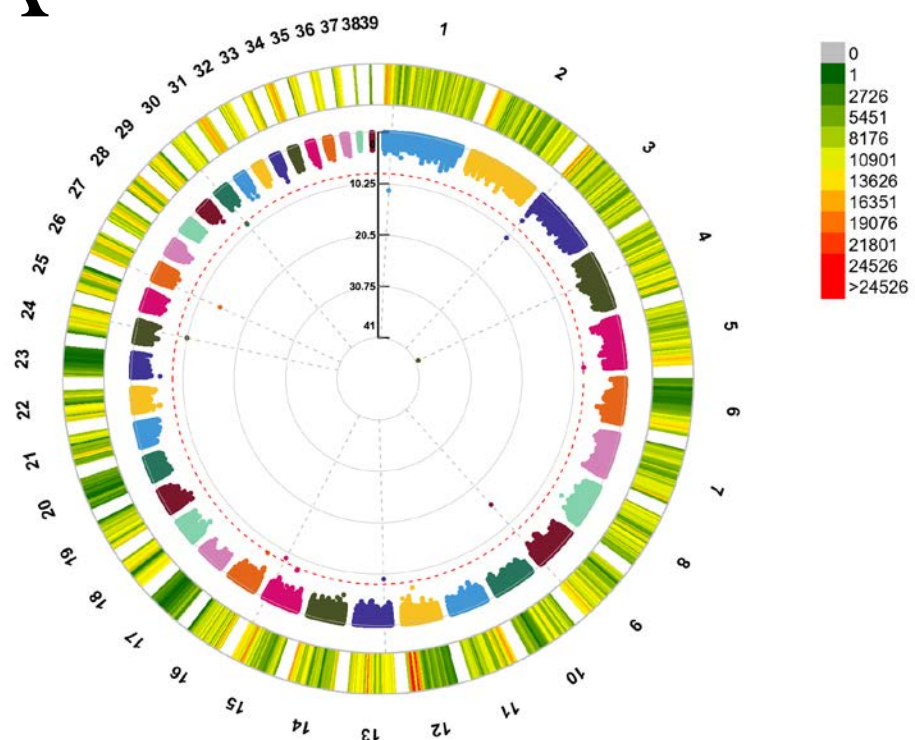

B

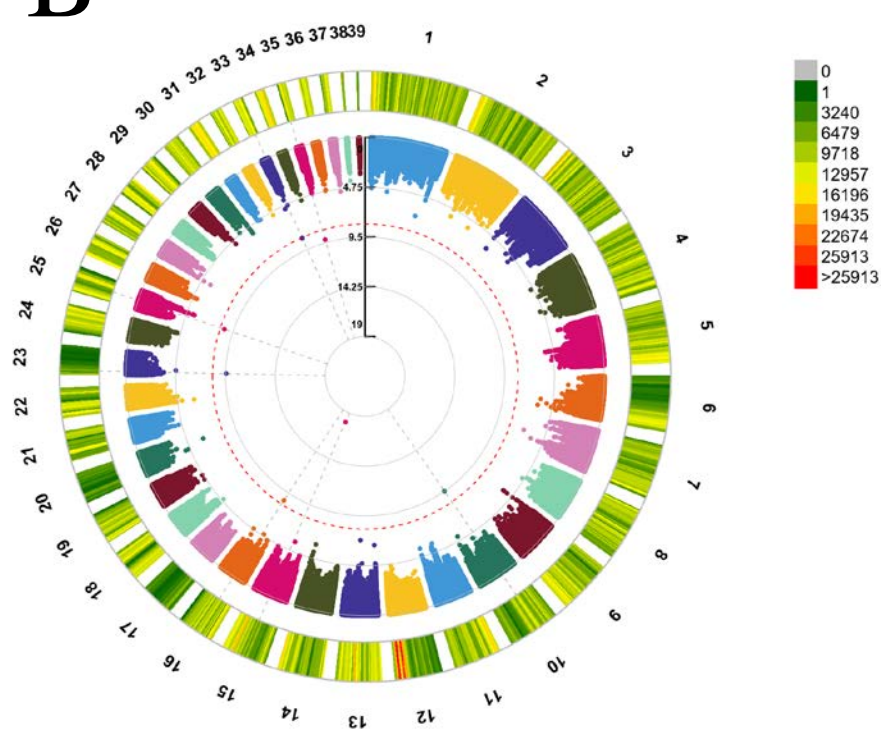

C

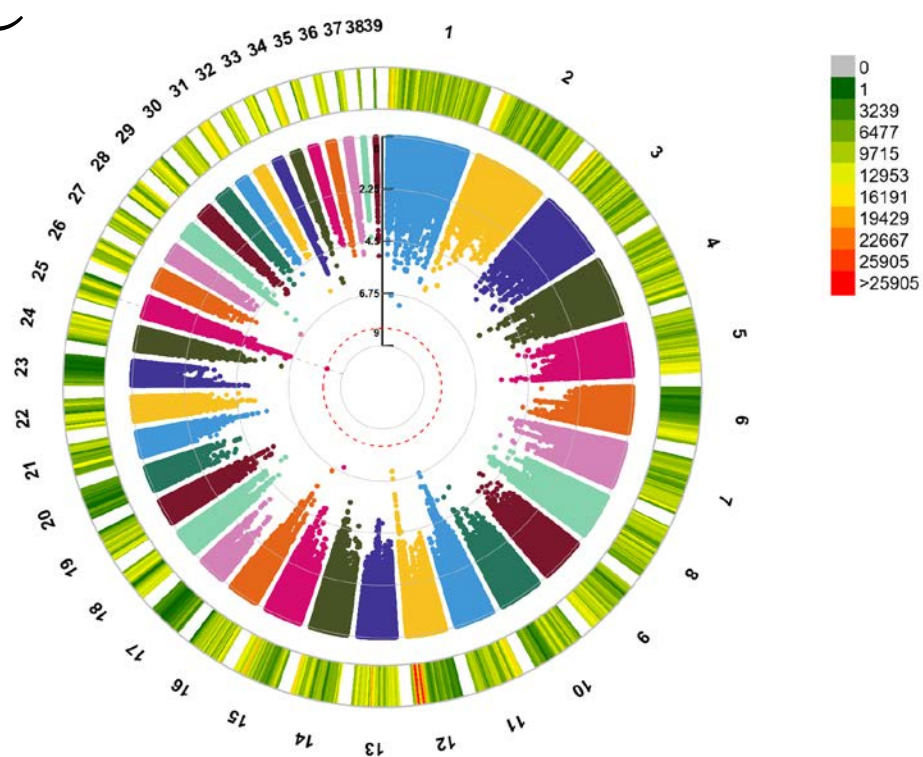

D

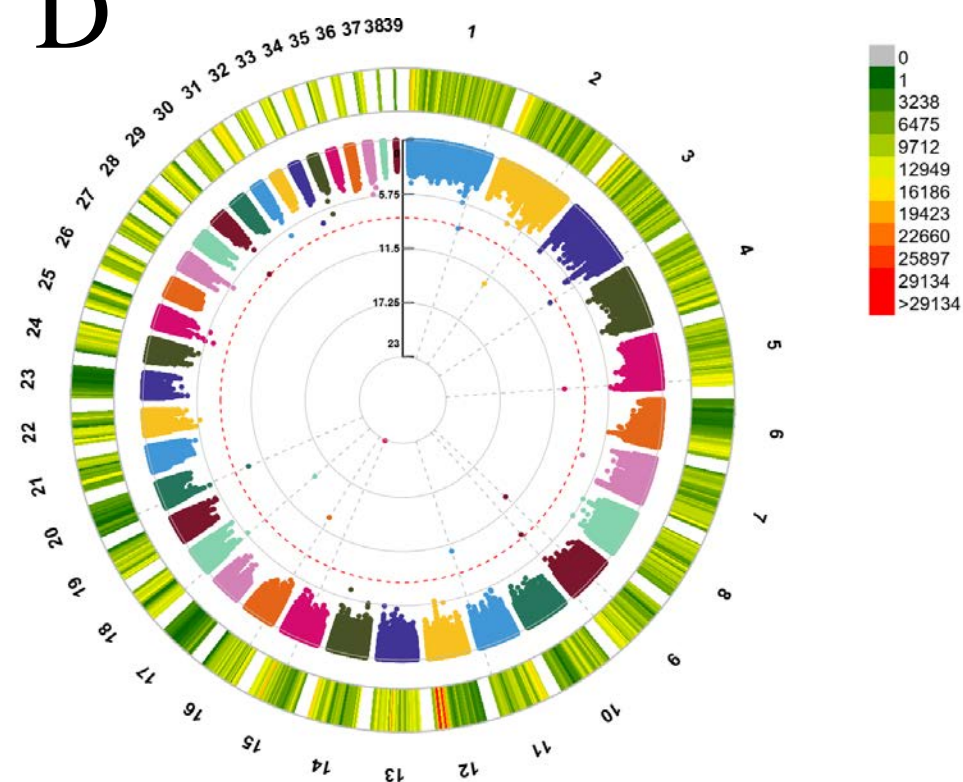

E

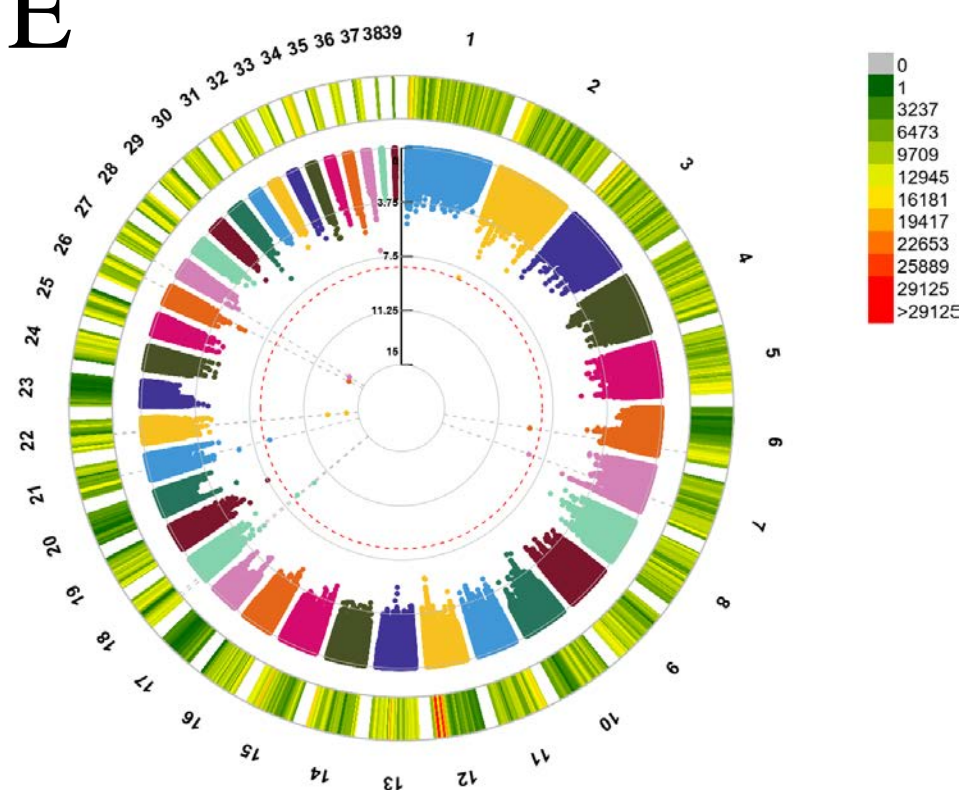

F

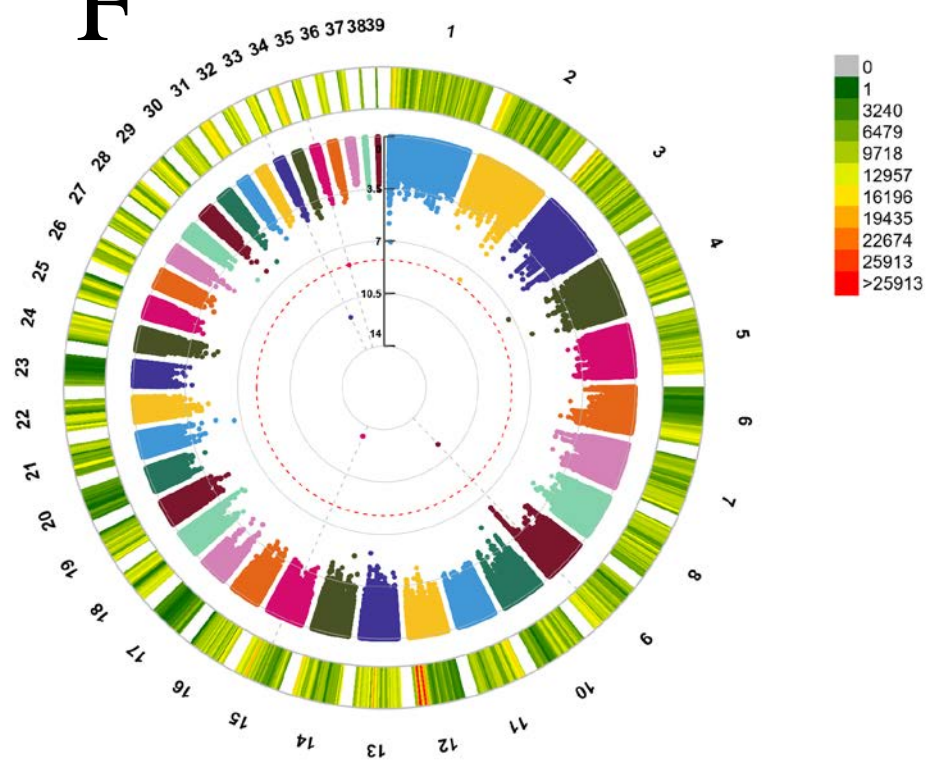

G

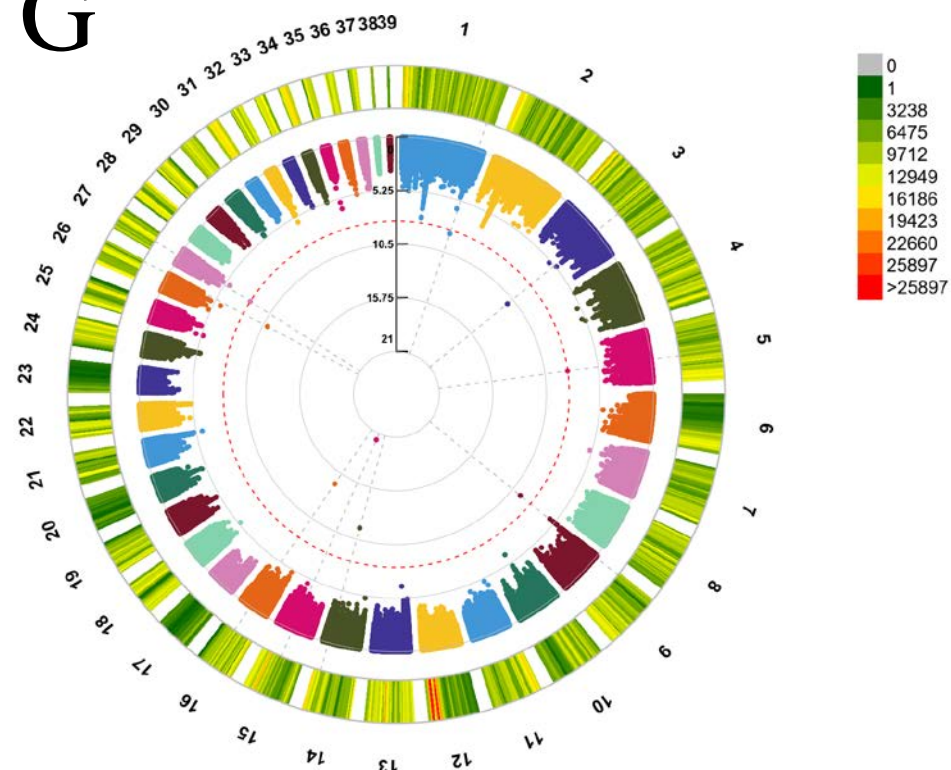

Supplement: Supplementary file 1 — Additional file 1: Fig. S1. The Manhattan plots of various phenotypes of Sichuan white geese. A-G represents the phenotypes of Sichuan white geese as follows: 9-20 wk geese feed to meat ratio, carcass keel bone length, chest meat pH, keel bone length, leg circumference, feet weight, and tibia length. [file 40104_2023_944_MOESM1_ESM.pdf]
