# Supplementary material for: Insights into genetic diversity and phenotypic variations in domestic geese through comprehensive population and pan-genome analysis
Source: J Anim Sci Biotechnol. 2023 Nov 24;14:150. doi: 10.1186/s40104-023-00944-y (PMC10675864; doi:10.1186/s40104-023-00944-y)
Supplement: Supplementary file 2 — Additional file 2: Fig. S2. GO enrichment analysis results of the shell genes. The GO terms were related to biological process (A), cellular component (B), and molecular function (C). [file 40104_2023_944_MOESM2_ESM.pdf]

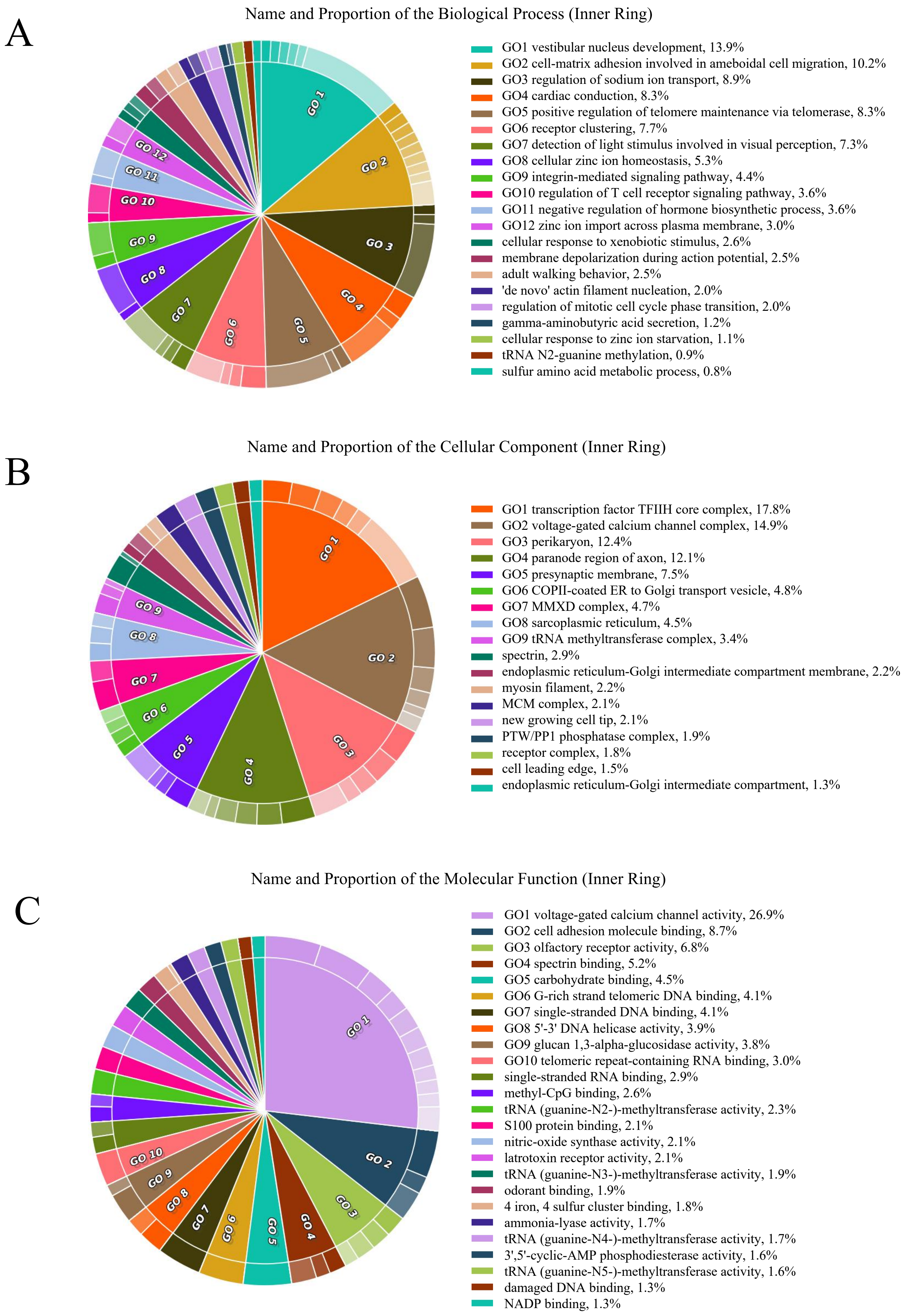
A
Name and Proportion of the Biological Process (Inner Ring)
GO1 vestibular nucleus development, 13.9%
GO2 cell-matrix adhesion involved in ameboidal cell migration, 10.2%
GO3 regulation of sodium ion transport, 8.9%
GO4 cardiac conduction, 8.3%
GO5 positive regulation of telomere maintenance via telomerase, 8.3%
GO6 receptor clustering, 7.7%
GO7 detection of light stimulus involved in visual perception, 7.3%
GO8 cellular zinc ion homeostasis, 5.3%
GO9 integrin-mediated signaling pathway, 4.4%
GO10 regulation of T cell receptor signaling pathway, 3.6%
GO11 negative regulation of hormone biosynthetic process, 3.6%
GO12 zinc ion import across plasma membrane, 3.0%
cellular response to xenobiotic stimulus, 2.6%
membrane depolarization during action potential, 2.5%
adult walking behavior, 2.5%
'de novo' actin filament nucleation, 2.0%
regulation of mitotic cell cycle phase transition, 2.0%
gamma-aminobutyric acid secretion, 1.2%
cellular response to zinc ion starvation, 1.1%
tRNA N2-guanine methylation, 0.9%
sulfur amino acid metabolic process, 0.8%
B
Name and Proportion of the Cellular Component (Inner Ring)
GO1 transcription factor TFIIH core complex, 17.8%
GO2 voltage-gated calcium channel complex, 14.9%
GO3 perikaryon, 12.4%
GO4 paranode region of axon, 12.1%
GO5 presynaptic membrane, 7.5%
GO6 COPII-coated ER to Golgi transport vesicle, 4.8%
GO7 MMXD complex, 4.7%
GO8 sarcoplasmic reticulum, 4.5%
GO9 tRNA methyltransferase complex, 3.4%
spectrin, 2.9%
endoplasmic reticulum-Golgi intermediate compartment membrane, 2.2%
myosin filament, 2.2%
MCM complex, 2.1%
new growing cell tip, 2.1%
PTW/PP1 phosphatase complex, 1.9%
receptor complex, 1.8%
cell leading edge, 1.5%
endoplasmic reticulum-Golgi intermediate compartment, 1.3%
C
Name and Proportion of the Molecular Function (Inner Ring)
GO1 voltage-gated calcium channel activity, 26.9%
GO2 cell adhesion molecule binding, 8.7%
GO3 olfactory receptor activity, 6.8%
GO4 spectrin binding, 5.2%
GO5 carbohydrate binding, 4.5%
GO6 G-rich strand telomeric DNA binding, 4.1%
GO7 single-stranded DNA binding, 4.1%
GO8 5'-3' DNA helicase activity, 3.9%
GO9 glucan 1,3-alpha-glucosidase activity, 3.8%
GO10 telomeric repeat-containing RNA binding, 3.0%
single-stranded RNA binding, 2.9%
methyl-CpG binding, 2.6%
tRNA (guanine-N2-)-methyltransferase activity, 2.3%
S100 protein binding, 2.1%
nitric-oxide synthase activity, 2.1%
latrotoxin receptor activity, 2.1%
tRNA (guanine-N3-)-methyltransferase activity, 1.9%
odorant binding, 1.9%
4 iron, 4 sulfur cluster binding, 1.8%
ammonia-lyase activity, 1.7%
tRNA (guanine-N4-)-methyltransferase activity, 1.7%
3',5'-cyclic-AMP phosphodiesterase activity, 1.6%
tRNA (guanine-N5-)-methyltransferase activity, 1.6%
damaged DNA binding, 1.3%
NADP binding, 1.3%
