# Supplementary material for: Insights into genetic diversity and phenotypic variations in domestic geese through comprehensive population and pan-genome analysis
Source: J Anim Sci Biotechnol. 2023 Nov 24;14:150. doi: 10.1186/s40104-023-00944-y (PMC10675864; doi:10.1186/s40104-023-00944-y)
Supplement: Supplementary file 3 — Additional file 3: Fig. S3. Summary of pan-genome and transcriptome studies in the present study. [file 40104_2023_944_MOESM3_ESM.pdf]

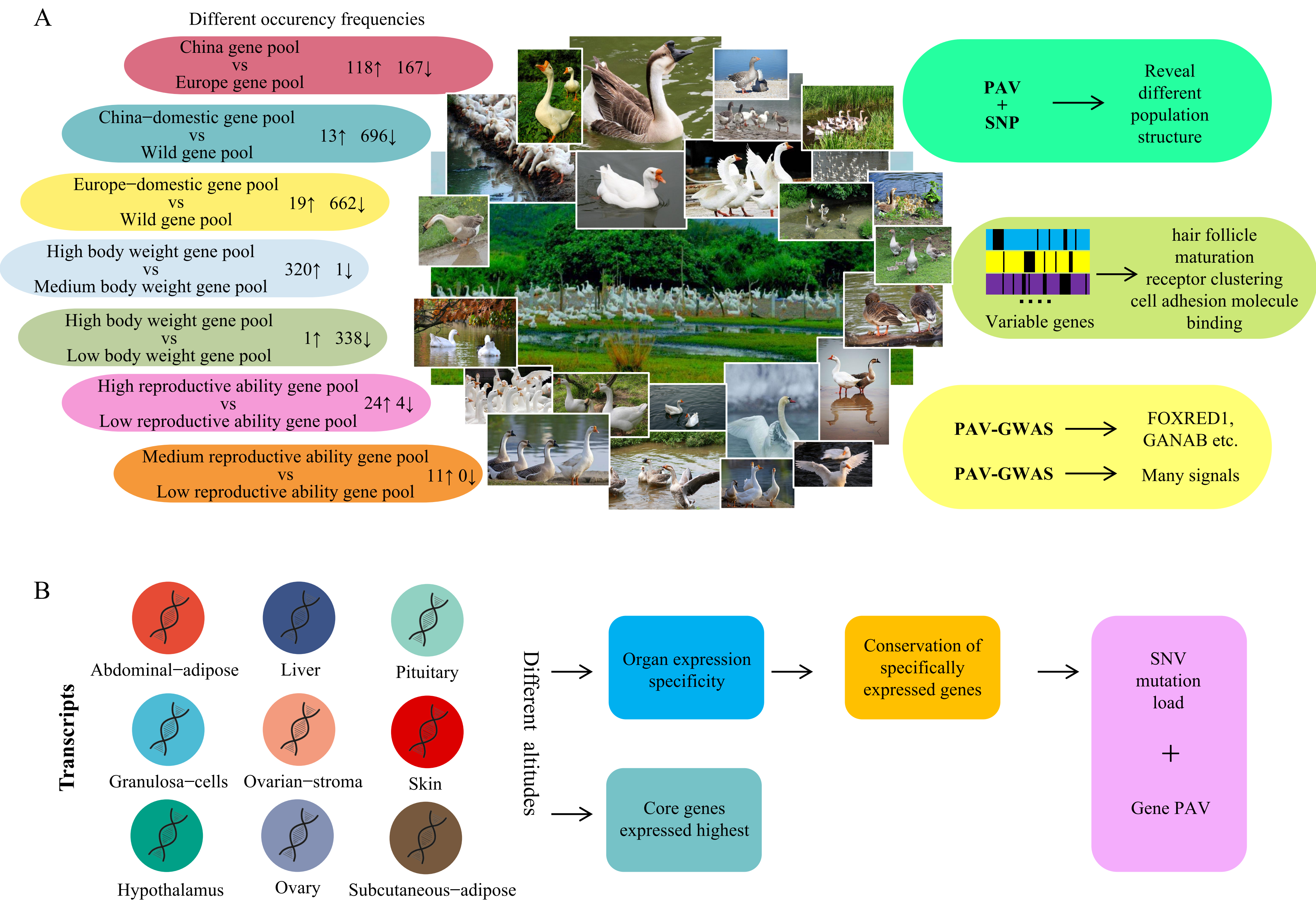
A
Different occurency frequencies
China gene pool vs Europe gene pool 118↑ 167↓
China−domestic gene pool vs Wild gene pool 13↑ 696↓
Europe−domestic gene pool vs Wild gene pool 19↑ 662↓
High body weight gene pool vs Medium body weight gene pool 320↑ 1↓
High body weight gene pool vs Low body weight gene pool 1↑ 338↓
High reproductive ability gene pool vs Low reproductive ability gene pool 24↑ 4↓
Medium reproductive ability gene pool vs Low reproductive ability gene pool 11↑ 0↓
PAV + SNP
Reveal different population structure
Variable genes
hair follicle maturation
receptor clustering
cell adhesion molecule binding
PAV-GWAS
FOXRED1, GANAB etc.
PAV-GWAS
Many signals
B
Transcripts
Abdominal−adipose
Liver
Pituitary
Granulosa−cells
Ovarian−stroma
Skin
Hypothalamus
Ovary
Subcutaneous−adipose
Different altitudes
Organ expression specificity
Conservation of specifically expressed genes
SNV mutation load
+
Gene PAV
Core genes expressed highest
